# Monoterpenes and Their Derivatives—Recent Development in Biological and Medical Applications

**DOI:** 10.3390/ijms21197078

**Published:** 2020-09-25

**Authors:** Mariola Zielińska-Błajet, Joanna Feder-Kubis

**Affiliations:** Faculty of Chemistry, Wrocław University of Science and Technology, Wybrzeże Wyspiańskiego 27, 50-370 Wrocław, Poland

**Keywords:** geraniol, thymol, myrtenal, pinene, camphor, borneol, biological activity, antiviral activity, analgesic activity, anti-inflammatory activity

## Abstract

Monoterpenes, comprising hydrocarbons, are the largest class of plant secondary metabolites and are commonly found in essential oils. Monoterpenes and their derivatives are key ingredients in the design and production of new biologically active compounds. This review focuses on selected aliphatic, monocyclic, and bicyclic monoterpenes like geraniol, thymol, myrtenal, pinene, camphor, borneol, and their modified structures. The compounds in question play a pivotal role in biological and medical applications. The review also discusses anti-inflammatory, antimicrobial, anticonvulsant, analgesic, antiviral, anticancer, antituberculosis, and antioxidant biological activities exhibited by monoterpenes and their derivatives. Particular attention is paid to the link between biological activity and the effect of structural modification of monoterpenes and monoterpenoids, as well as the introduction of various functionalized moieties into the molecules in question.

## 1. Introduction

**Figure 1:**
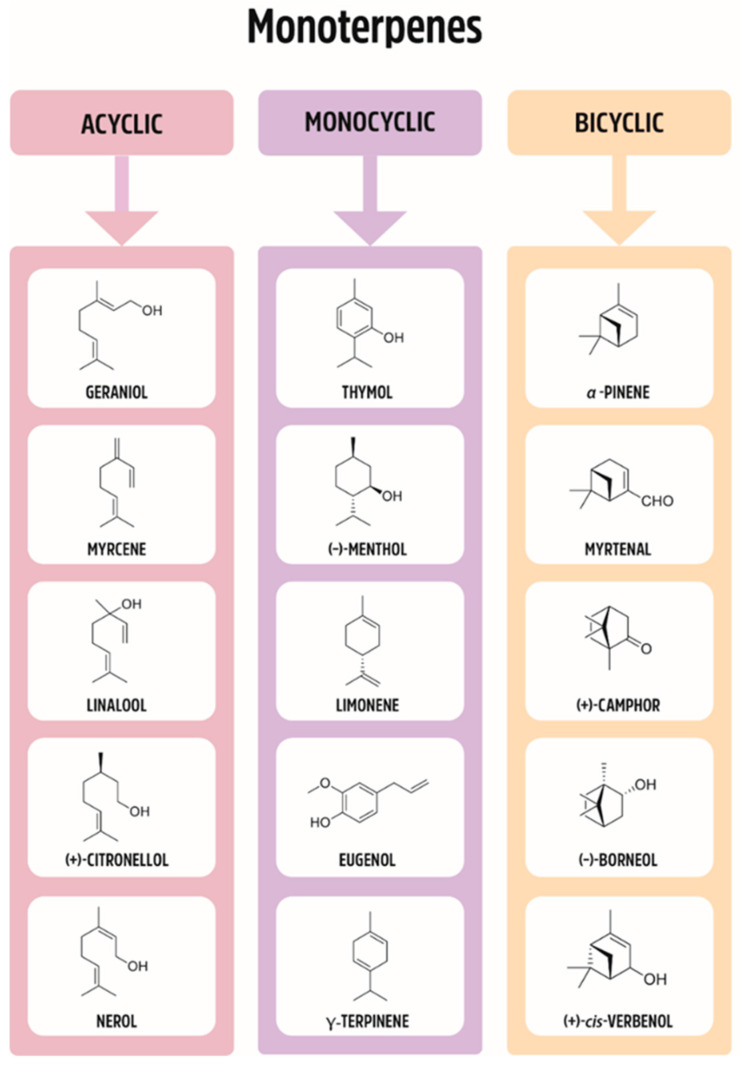
Division of monoterpenes (acyclic, monocyclic, bicyclic).

Plants have been used for medical purposes for centuries. As such, they have been studied scientifically and traded commercially. Monoterpenes belong to a large and diverse group of naturally occurring compounds. The basic structure of monoterpenes, or monoterpenoids, consists of two linked isoprene units; some of the representative compounds are given in Figure 1. They might be cyclized and oxidized in a variety of ways. Due to the low molecular weight, many of them exist in the form of essential oils. The numbers of monoterpenes have antimicrobial, anti-inflammatory, antioxidant, antipruritic, hypotensive, and analgesic pharmacological properties.

**Figure 2:**
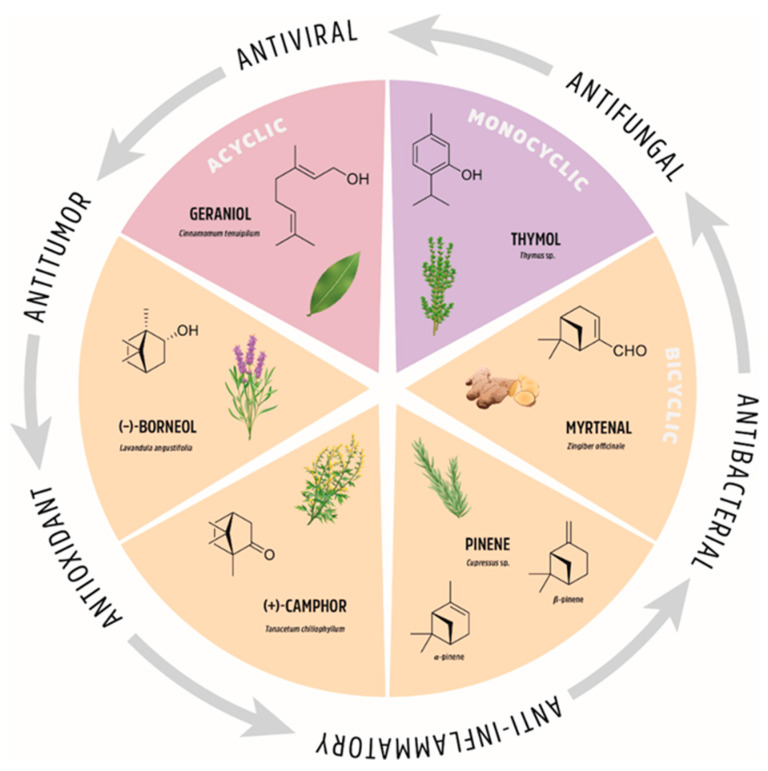
The natural origin of monoterpenes and their biological applications.

Functionalization of natural compounds exhibiting native biological activity is one of the most efficient approaches for the synthesis of biologically active substances in medicinal chemistry. This explains, inter alia, why the studies of terpenes and their derivatives are interesting and relevant. Consequently, there is growing body of literature regarding new modifications to these fascinating natural compounds and the corresponding biological and medical applications. Despite the importance of this class of compounds, there are limited reviews on the biological and pharmacological properties of monoterpene derivatives [1,2,3,4,5,6]. Hence, we considered it appropriate to provide a review of selected monoterpene derivatives and their diverse therapeutic activities. We pay particular attention to the structural changes in monoterpenes and the introduction of special groups that increase the biological activity of the studied derivatives. We focus on structural modifications of geraniol (representative of acyclic monoterpenes), thymol (representative of monoterpene monocycles), myrtenal, pinene, camphor, and borneol (representatives of monoterpene bicycles); see Figure 2 for an overview. We present those monoterpene derivatives that exhibit several important biological properties; they are often multi-functional or exhibit interesting biological selectivity in their activity.

## 2. Acyclic Monoterpene Derivatives

### Geraniol

Geraniol, *trans*-3,7-dimethyl-2,6-oktadien-1-ol, is a primary, acyclic, doubly unsaturated terpene alcohol with a characteristic flowery, rose-like odor. This monoterpene is extracted from the essential oils of several aromatic plants including *Cinnamomum tenuipilum* Kosterm., *Valeriana officinalis* subsp. *collina* (Wallr.) Nyman, *Phyla scaberrima* (Juss. ex Pers.) Moldenke, and others [7]. The limited production of geraniol via plant extraction cannot satisfy a great demand from the flavor, fragrance, and other industries, which can only be met by the production utilizing biotechnological processes. Geraniol possesses numerous beneficial medicinal properties, including antioxidant, anti-inflammatory, antimicrobial, antitumor, hepatoprotective, cardioprotective, and neuroprotective [8,9].

**Figure 3:**
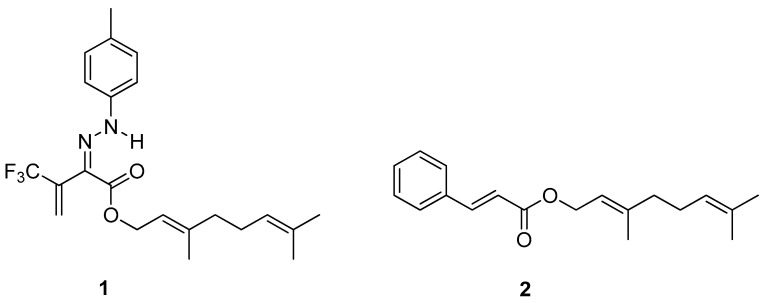
Ester derivatives of naturally occurring geraniol.

A series of 3-oxo-2-tolylhydrazinylidene-4,4,4-trifluorobutanoates containing higher or natural alcohol moieties in their structures were designed and synthesized in response to the need for effective and selective inhibitors of carboxylesterase (CES) [10]. The ester derivative of naturally occurring geraniol is one of them and it proved to be a highly active and selective inhibitor of hCES2, blocking the enzyme within the nanomolar range (IC_50_ to ca. 5 nM). Geranyl (2*Z*)-4,4,4-trifluoro-2-[2-(4-methylphenyl) hydrazinylidene]-3-oxobutanoate **1** (Figure 3) show radical-scavenging properties and low acute toxicity, which is why the tested ester derivative of geraniol **1** has a great potential as a biomedicinal inhibitor.

Geranyl cinnamate ester **2** (Figure 3), tested on *Staphylococcus aureus* and *Escherichia coli,* exhibited excellent antimicrobial (Gram-positive and Gram-negative bacteria) activity, which might be useful in biotechnology. The inhibition zone for **2** demonstrated the synergic effects of two combined natural substrates: geraniol and cinnamic acid.

**Figure 4:**
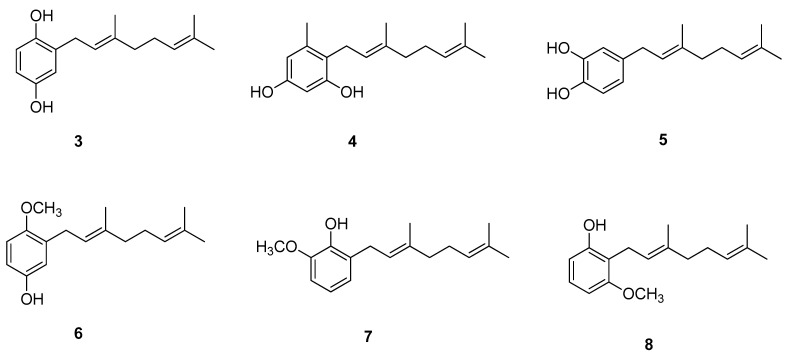
Geraniol-based phenols/methoxyphenols.

Chavez et al. [11] showed interesting results regarding biologically active geraniol derivatives. A large series of geranylated phenol/methoxyphenol compounds were synthesized to test them in vitro as inhibitor agents of mycelial growth of *Phytophthora cinnamomi*. It turned out that geranylated derivatives containing two hydroxyl groups attached to the aromatic ring, or one of them exchanged by a methoxy group, are the most efficient inhibitors. Some selected structures from the set studied by Chavez et al. are shown in Figure 4 (compounds **3–8**). Importantly, the activity of those derivatives is as effective as that of Metalaxil^®^, a commonly known commercial fungicide, used as an inhibitor of mycelial growth of *P. cinnamomi*. Some of the discussed compounds are also active against *Botrytis cinerea*, which makes them possibly useful for biotechnological purposes since they may control the infection with more than one pathogen.

**Figure 5:**
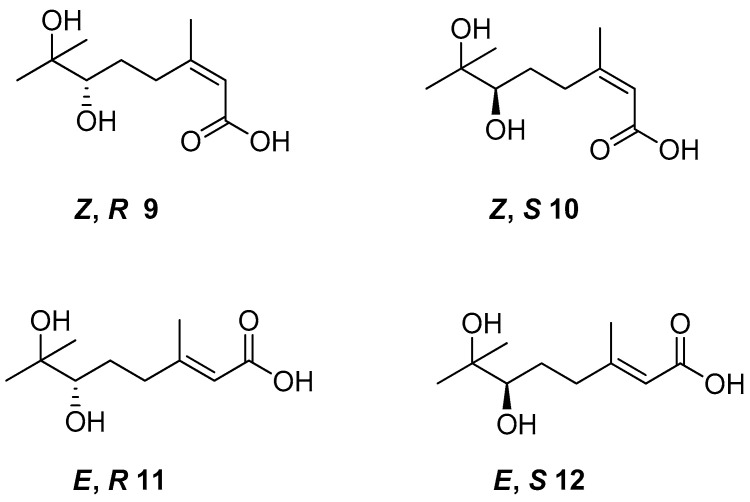
The isomers of 6,7-dihydroxy-3,7-dimethyloct-2-enoic acid derivatives of geraniol.

*Cis*- and *trans*-geraniol as raw material was used for the synthesis of four isomers of 6,7-dihydroxy-3,7-dimethyloct-2-enoic acid **9–12** (Figure 5) to obtain monoterpene derivatives with antifungal activity. The in vivo bioassay results showed that chiral acid (*Z*,*S*)—**10** demonstrated promising results as an antifungal agent. The tests confirmed 80–100% inhibitory rates of (*Z*,*S*)-6,7-dihydroxy-3,7-dimethyloct-2-enoic acid against *Pseudoperonospora cubensis*, *Erysiphe graminis*, *Puccinia sorghi,* and *Colletotrichum gloeosporioides* at a concentration of 400 µM.

**Figure 6:**
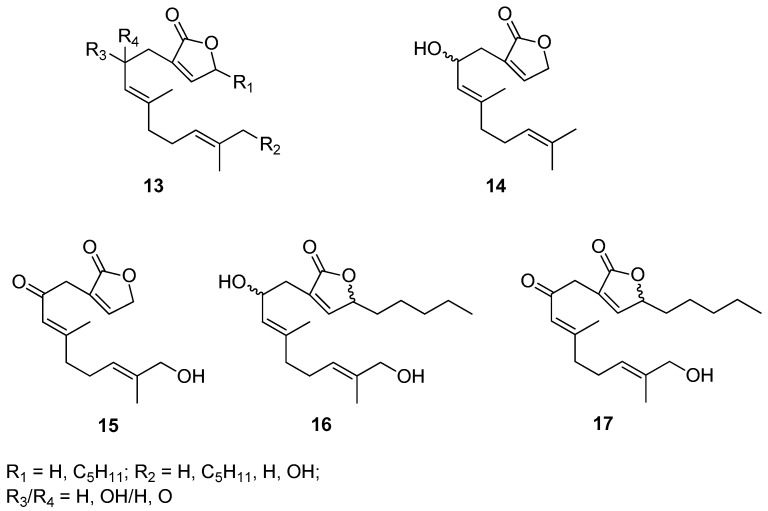
Selected examples of geraniol-butanolide hybrid compounds.

Biofouling that occurs on the surface of submerged objects, which causes technical and economic problems mainly in the marine industry, but also in many biotechnological processes, is a stimulus to the design and synthesis of effective, safe, and at the same time environmentally benign antifouling compounds. Motivated by these challenges Takamura’s research group designed and synthesized a hybrid of molecules containing geraniol and butanolide moieties [12]. A set of prepared geraniol derivatives consists of eight various molecules **13**, some representatives of this series (**14–17**) are included in Figure 6. Biological evaluation of presented geraniol-butanolide hybrid compounds **13** proved that those molecules exhibit antifouling activity against the cypris larvae of the barnacle *Balanus amphitrite* (Darwin, 1854), having the values of EC_50_ within the range of 0.30–1.31 μM. Since most of those compounds, **13** show low or no toxicity geraniol derivatives might be successfully applied as antifouling agents.

## 3. Monoterpene Monocyclic Derivatives

### Thymol

Thymol (2-isopropyl-5-methylphenol) is a natural phenolic monoterpenoid extracted primarily from *Thymus* species (*Trachyspermum ammi* (L.) Sprague) and other plant sources such as *Baccharis Grisebachii* Hieron. and *Centipeda minima* (L.) A. Br. & Asch. and widely used in pharmaceutical and food preservative applications. It is also obtained by various methods at industrial scale from *m*-cresol, or *p*-cymene and piperitone [13,14,15]. Thymol, a component of naturally occurring essential oils, exhibits multiple biological activities [5,6]. It is antimicrobial [16,17,18,19] and anti-inflammatory activity [20,21], which means that it is suitable to heal wounds [22], antioxidative [23,24], anticancer activity against human gastric cancer cells [25]. With its minimal potential toxicity, it shows a promise as a novel botanical pesticide [26].

The derivatives with the thymol-based scaffolds have gained much attention owing to their antibacterial, antifungal, anti-inflammatory, anticancer, antitumor, anti-HIV, antiviral, antipyretic, anticonvulsant, and antidepressant properties.

**Figure 7:**
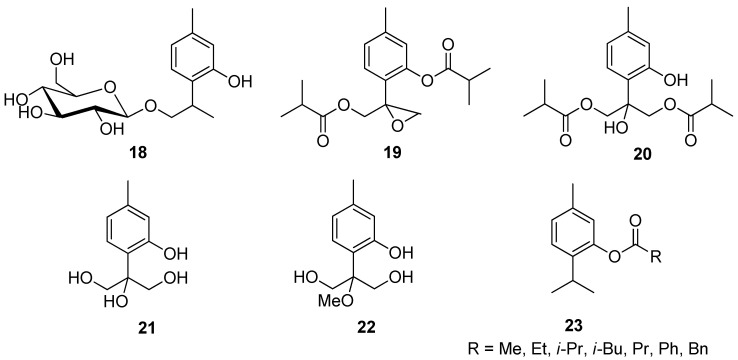
Representative thymol’s derivatives with antimicrobial and antibacterial activities.

Zhao et al. isolated different thymol derivatives **18**–**22** from *Inula hupehensis* (Ling) Ling and tested them for their activity against the *Staphylococcus aureus*, *Methicillin*-resistant *S. aureus*, and *Escherichia coli*. It was found by the authors that 8-hydroxy-9,10-diisobutyloxy thymol **20** is the most active at MIC values 62.3, 62.8, and 250 μM and it shows inhibitory activities against *Rhizoctonia solani*, *Phytophthora melonis,* and *Peronophythora litchi* with EC_50_ of 157, 180, and 141 μM (Figure 7) [27].

Mathela and co-workers determined the antibacterial activity of thymol and thymol-derived esters **23** against Gram-positive *Streptococcus mutans*, *S. aureus*, *Bacillus subtilis*, *S. epidermidis*, and one Gram-negative *E. coli* [28]. Thymol ester derivatives show the most enhanced activity against Gram-positives. 2-Isopropyl-5-methylphenyl acetate **23a** and 2-isopropyl-5-methylphenyl isobutyrate **23c** were found to be more effective than thymol and all other esters against *S. mutans* (MIC = 11.7 and 93.7 μM), *B. subtilis* (MIC = 11.7 and 46.8 μM), and *S. epidermidis* (MIC = 11.7 and 46.8 μM), whereas 2-isopropyl-5-methylphenyl propionate **23b** was found to be more active than thymol only against *B. subtilis* (MIC = 46.8 μM) and *S. epidermidis* (MIC = 46.8 μM) (Figure 7). Another study conducted by Chauhan et al. determined the antifungal effectiveness of thymol ester derivatives against the common soilborne plant pathogenic fungus *R. solani*. Thymol acetate **23a** was found to be a highly effective plant-based fungicide [29].

**Figure 8:**
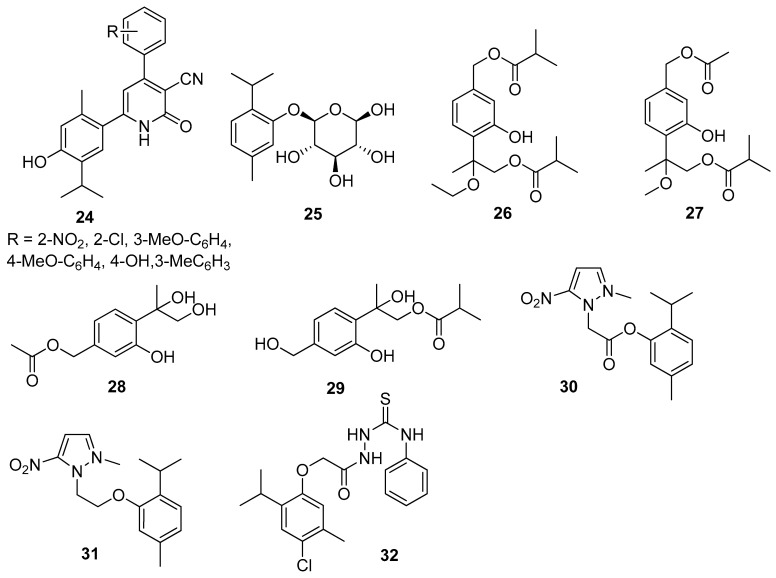
Representative thymol derivatives with antibacterial activity.

Nagle et al. synthesized thymol derivatives containing pyridine moieties **24** and assessed their antioxidant and antimicrobial properties. The 2-pyridone derivatives exhibit good antifungal activity and a better bacterial inhibition against *E. coli* and *S. aureus* species than thymol. All derivatives are also better antioxidants than the parent compound [30]. Thymol-*β*-D-glucopyranoside **25** exhibits anticampylobacter activity (Figure 8) [31].

Tan and co-worker isolated two new thymol derivatives, 7,9-diisobutyryloxy-8-ethoxythymol **26** and 7-acetoxy-8-methoxy-9-isobutyryloxythymol **27**, from fresh *Ageratina adenophora* (Spreng.) R.M.King & H.Rob. roots and tested them for their in vitro antibacterial activity toward three Gram-positive and two Gram-negative bacterial strains. Thymol derivatives **26** and **27** show in vitro bacteriostatic activity toward three Gram-positive bacteria strains (Figure 8) [32].

Liu’s group isolated a monoterpenoid, 7-acetyl-8,9-dihydroxy thymol **28** together with 7,8-dihydroxy-9-butyryl thymol **29** (Figure 8) from dried flower buds of *Lonicera japonica* Thunb. Both derivatives exhibit significant antibacterial impact on *S. aureus*, *E. coli*, *M. luteus*, and *B. cereus* with IC_50_ values ranging from 27.64 to 128.58 μM [33].

Other research teams synthesized metronidazole ester **30** and ether **31** with thymol and investigated their in vitro antibacterial activity on *Helicobacter pylori* (the ATCC 26695 and P12) and *Clostridium* (*C. perfringens*) strains. Both compounds demonstrated good activity against *H. pylori* strain. It is worth noting that the ether derivative (**31**) is more active than the ester (Figure 8) [34].

El-Miligy et al. focused their attention on the preparation of various thymol hybrid molecules with dual antimicrobial and insecticidal activities free of the irritation side effect from the blocked phenolic hydroxyl group. The targeted compounds were designed to link the biologically active thymol scaffold with different five-membered heterocyclic moieties like 1,3,4-oxadiazole, 1,3,4-thiadiazole, 1,2,4-triazole, thiazole, and 4-thiazolidinone through different spacers. All newly synthesized compounds were evaluated in vitro for their antibacterial activity against *B. subtilis*, *S. aureus*, *E. coli,* and *P. aeruginosa* and tested for their in vitro antifungal activity against *C. albicans*. Only 2-[2-(4-chloro-2-isopropyl-5-methylphenoxy)acetyl]-*N*-phenylhydrazine-1-carbothioamide **32** turned out to have dual antimicrobial activity against *S. aureus*, *B. subtilis* and insecticidal activity against *Tribolium castaneum* (*Herbst*) and *Sitophilus oryzae* (L.) (Figure 8) [35].

**Figure 9:**
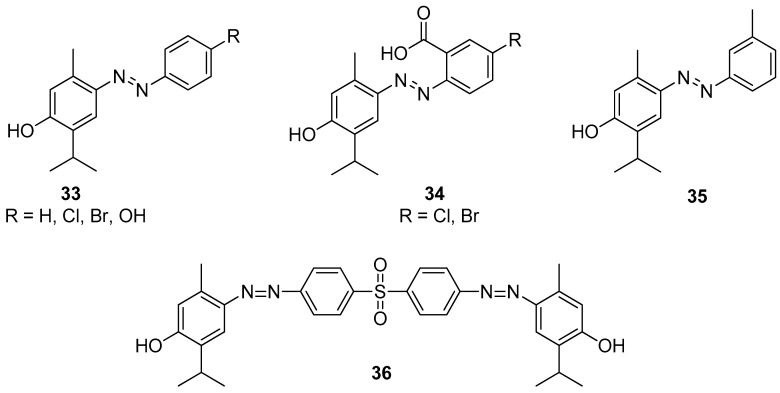
Selected aryl-azo-thymol derivatives with significant antibacterial and antifungal activities.

Swain et al. [36] synthesized 12 substituted aryl-azo-thymol derivatives. Antimicrobial activities were evaluated by agar-well diffusion against isolated MRSA, ESBL-producing pathogenic bacteria (*S. aureus*, *E. coli*, *K. pneumoniae*, *P. aeruginosa*), and antifungal resistant fungi (*A. fumigatus*, *C. albicans*), in vitro. Among all derivatives, compounds **33–35** (Figure 9) have significant antibacterial and antifungal activities with MICs ranging from 40 to 80 μM. The docking scores of derivatives were −8.27 to −11.44 kcal/mol, against four bacterial targets and −9.45 to −12.49 kcal/mol against two fungal targets. Thus, as demonstrated by in vitro and silico studies, thymol derivatives are efficient in control over MRSA, ESBL-producing bacteria. A study conducted by Kaur et al. suggested that the compound 4,4′-((sulfonylbis(4,1-phenylene))bis(diazene-2,1-diyl))bis(2-isopropyl-5-methylphenol) **36** shows moderate antifungal activity against *A. niger*, *T. rubrum* and *C. glabrata* (Figure 9) [37].

**Figure 10:**
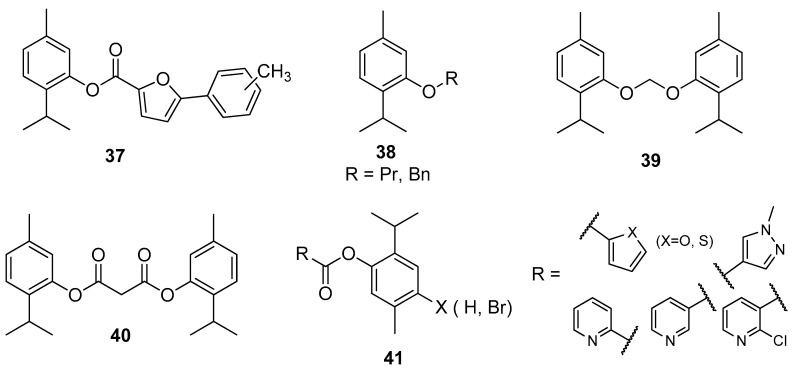
Selected thymol derivatives with significant antifungal activities.

Nishida and co-workers synthesized thymol derivatives **37**–**40** for antifungal potency against *Aspergillus niger*, *Aspergillus oryzae*, *Fusarium oxysporum*, and *Alternaria alternate*. The antitumor tests showed that 2-isopropyl-5-methylphenyl 5-phenylfuran-2-carboxylate **37** exhibits promising activity against Bel-7402 and KB. Dithymyl ethylene ether thymol derivative **39** shows the highest antifungal activity against *A. niger* (Figure 10) [38].

Wang et al. designed and synthesized thymol esters with different heterocyclic units **41** (Figure 10) and tested them for their in vitro antifungal activity against five plant pathogenic fungi (*Alternaria solani*, *Botrytis cinerea*, *Fusarium oxysporum*, *Pyricularia oryzae*, and *Rhizoctonia solani*) by the mycelium growth rate method. Some synthetic esters show good to excellent antifungal activity, especially with furan, thiophene, and pyridine unit. The presence of a bromine atom on the *para* position of benzene moiety increased the antifungal activity [39].

**Figure 11:**
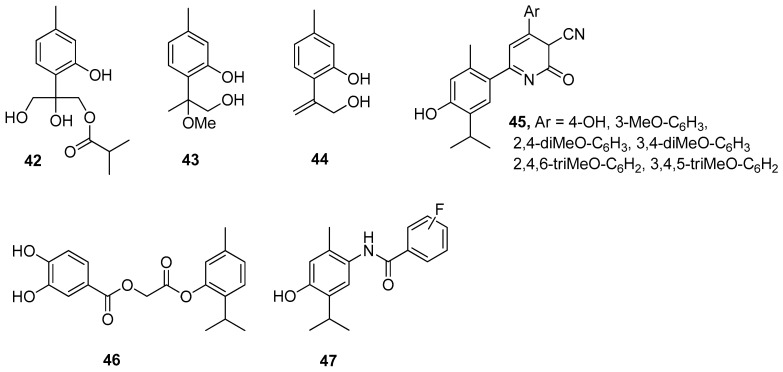
Selected thymol derivatives with antioxidant activities.

The antioxidant activity of thymol is attributed to its phenolic structure and its redox properties, which play an essential role in the adsorbing and neutralizing of free radicals or decomposing peroxides. A wide range of thymol derivatives was explored for structure-radical scavenging activity. By comparing the structural properties of thymol derivatives, their antioxidant activity was explained by the formation of an intramolecular hydrogen bond. Three thymol derivatives, 8,10-dihydroxy-9-isobutyryloxythymol **42**, 8-methoxy-9-hydroxythymol **43**, and 8,9-dehydro-10-hydroxythymol **44**, were isolated from Zataria multiflora extract. The antioxidant activity of these compounds was evaluated with the 2,2-diphenyl-1-picrylhydrazyl (DPPH) assay. Compound **44** has higher antioxidant potential with IC_50_ value of 62.5 μM and BDE = 79.2 kcal/mol. These results show that the presence of an unsaturated double bond is the main factor that determines the antioxidant activity of the investigated compounds (Figure 11) [40]. 

Nagle et al. [30] evaluated 1,2-dihydro-6-(4-hydroxy-5-isopropyl-2-methylphenyl)-2-oxo-4-arylpyridine-3-carbonitriles for their in vitro antioxidant and antimicrobial activity. Compounds **45** shows much better antioxidant activity than other derivatives (Figure 11).

Recently, Ashraf’s group has studied the substituted benzoic acid and cinnamic acid thymol moieties. They observed that 2-[5-methyl-2-propan-2-yl)phenoxy]-2-oxoethyl-3,4-dihydroxybenzoate **46** has a higher antioxidant potential (IC_50_ = 11.30 μM) in comparison to standard ascorbic acid (IC_50_ = 24.20 μM) (Figure 11) [41].

Bendre and co-worker synthesized a series of thymol-based benzamides **47** (Figure 11), structurally similar to paracetamol [42]. These compounds were subjected to antioxidant testing by DPPH assay. The results revealed that all the synthesized compounds exhibit notable antioxidant activity and also molecular docking studies indicated that they are good inhibitors of heme oxygenase-1.

**Figure 12:**
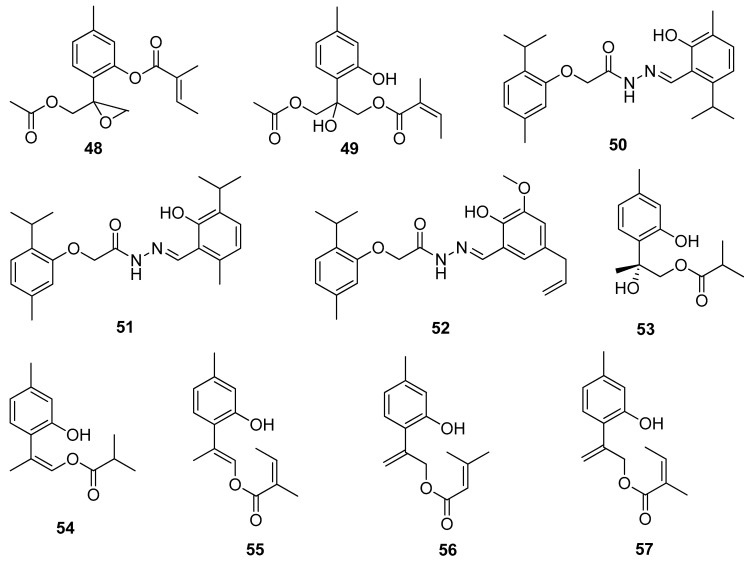
Thymol derivatives with anticancer activity.

A study conducted by Chen and co-workers evaluated the cytotoxic activity of various thymol derivatives isolated from the root of *Eupatorium cannabinum* subsp. *Asiaticum* Kitam. All compounds were tested in vitro against DLD-1, CCRF-CEM, HL-60, and P388D1 cell lines. The best cytotoxicity is exhibited by 9-acetoxy-8,10-epoxythymol 3-*O*-tiglate **48** (IC_50_ values of 0.02 ± 0.01, 1.02 ± 0.07, and 1.36 ± 0.12 μM) and 10-acetoxy-9-*O*-angeloyl-8-hydroxythymol **49** (IC_50_ values of 1.14 ± 0.16 and 2.63 ± 0.22, and 7.63 ± 0.94 μM) against the first three cell lines (Figure 12) [43].

Rajput et al. [44] synthesized thymol-based hydrazone derivatives **50**–**52** by the condensation of thymol with substituted 2-acetohydrazide of carvacrol and thymol. All compounds were screened for their antioxidant activity with DPPH assay and anticancer activity with SRB assay for the pancreas and colon. The molecular docking studies of all the synthesized derivatives were performed on the COX-2 protein enzyme. In the anticancer and antioxidant tests, thymol hydrazones indicate moderate activities. The molecular docking studies demonstrated excellent binding affinity towards the enzyme (Figure 12).

New thymol derivatives **53** and **54** (Figure 12) were isolated from the roots of *Eupatorium chinense* L. and tested in vitro for their cytotoxic activities against three cancer cell lines CNE 2, Caski, and HGC-27. The considerable cytotoxic effects against the three cancer cell lines of compound **53** were observed with the IC_50_ values ranging from 4.2 to 11.9 μM [45].

More recently, Shi and co-workers isolated a few thymol derivatives from the overground parts of *Eupatorium fortunei* Turcz. All compounds were screened for their cytotoxic activities against four human cancer cell lines using MTT assay. Compounds 9-*O*-angeloyloxy-8,9-dehydrothymol **55**, 9-(3-methyl-2-butenoyloxy)-8,10-dehydrothymol **56** and 9-*O*-angeloyl-8,10-dehydrothymol **57** (Figure 12) exhibit notable cytotoxicities with IC_50_ values 6.24–11.96 μM against MCF-7, HeLa, A549, and Hep G-2 cell lines. The preliminary research indicates that the presence of the methyl group at the C-1 position and the substituted group at C-9 in the structure of these thymol derivatives seem to be essential for the inhibition of the proliferation of four cancer cell lines [46].

**Figure 13:**
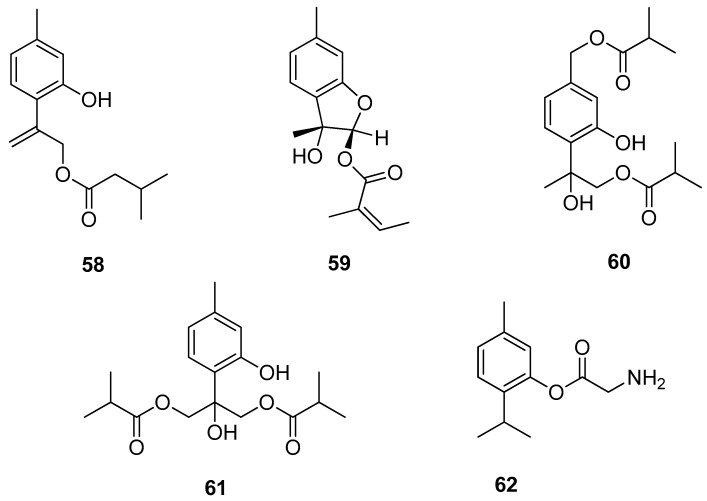
Thymol derivatives with anti-inflammatory activity.

Chen et al. [47] isolated several new compounds from the aerial part of *Eupatorium cannabinum* subsp. *asiaticum* Kitam. and assessed their antiinflammatory activities. 9-(3-Methylbutanoyl)-8,10-dehydrothymol **58** and eupatobenzofuran **59** exhibit fMLP/CB (formyl-l-methionyl-l-leucyl-l-phenylalanine/cytochalasin B)-induced elastase release with IC_50_ values ≤ 18.3 μM (Figure 13).

Various thymol derivatives were isolated from the aerial part of *Inula wissmanniana* Hand.-Mazz. All compounds were tested for their anti-inflammatory activities against LPS-induced NO production in RAW 264.7 macrophages. 8-Hydroxy-7,9-di-isobutyryloxythymol **60** and 7-hydroxy-8,9-bis(isobutyryloxy)thymol **61** show moderate activities, with IC_50_ values of 10.8 and 10.1 μM (Figure 13) [48]. Nesterkina et al. [49] synthesized glycine ester of mono-terpenoid. Compound 2-isopropyl-5-methylphenyl 2-aminoacetate **62** demonstrates both anti-inflammatory and analgesic activity (Figure 13).

**Figure 14:**
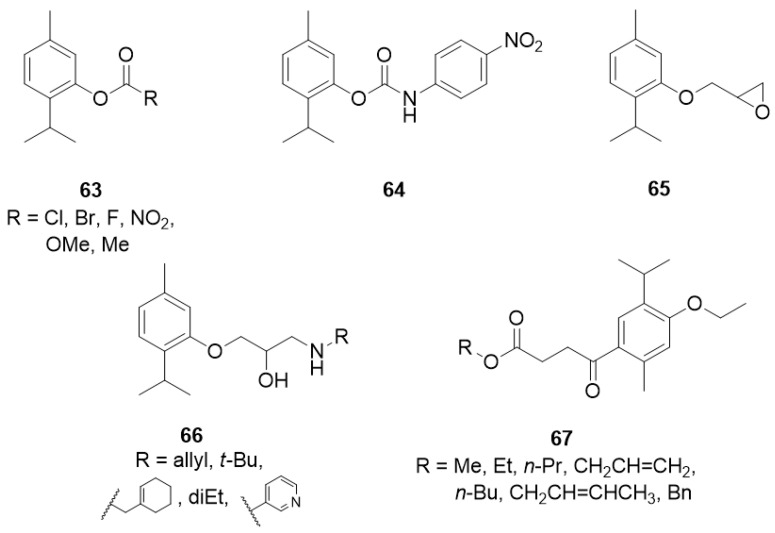
Thymol derivatives with inhibitory effects on some metabolic enzymes.

The aromatic esters of thymol **63** (Figure 14) were obtained, and their inhibition of the enzyme acetylcholinesterase, and larvicidal activity against *Aedes aegypti* was evaluated. The presence of a benzoate group containing electron-withdrawing substituents on the aromatic ring resulted in the inactivation of the compound against the mosquito larvae, while thymol 4-methoxybenzoate **63e** with electron-donating group exhibits much better activity [50]. 

Kurt et al. [51] synthesized a series of thymol derivatives with the carbamate moiety and tested their inhibitory effects on acetylcholinesterase (AChE) and butyrylcholinesterase (BuChE). Among the thymol derivatives, 2-isopropyl-5-methylphenyl (4-nitrophenyl)carbamate **64** (Figure 14) shows the strongest inhibition against AChE with an IC_50_ value of 2.50 μM. Moreover, insignificant cell death and nontoxicity at 0.07–10 μM in H4IIE hepatoma cell toxicity assay was found.

Another group of researchers synthesized novel oxypropanolamines containing thymol structure **65** and **66** and evaluated their inhibitory effects on *α*-glycosidase, cytosolic carbonic anhydrase I and II isoforms (hCA I and II), and acetylcholinesterase enzymes (AChE) (Figure 14). The results demonstrated that all compounds effectively inhibit *α*-glycosidase, hCA I and II, and AChE enzymes (with Ki values in the range of 63.85–851.05 μM for *α*-glycosidase, 1.11–17.34 μM and 2.97–17.83 μM for hCA I and hCA II, respectively, and 13.58–31.45 μM for AChE). Furthermore, the authors concluded that derivatives **66a**-**c** exhibited the most significant antibacterial activity on *A. baumannii*, *P. aeruginosa*, *E. coli,* and *S. aureus* strains among all the tested compounds [52].

Henry and co-workers synthesized seven new alkyl 4-oxobutanoate derivatives of thymol **67** and screened them for tyrosinase inhibitory activity using mushroom tyrosinase (Figure 14). Generally, the alkyl 4-oxobutanoate derivatives exhibit better inhibitory effects than the ethyl thymol 4-oxobutanoic acids. The data demonstrated the correlations between the structure of the alkyl side chain and tyrosinase inhibitory activity. The best results were obtained for esters containing three or four carbon atoms in the alkyl chain with IC_50_ values range from 102.3 to 191.4 μM [53].

**Figure 15:**
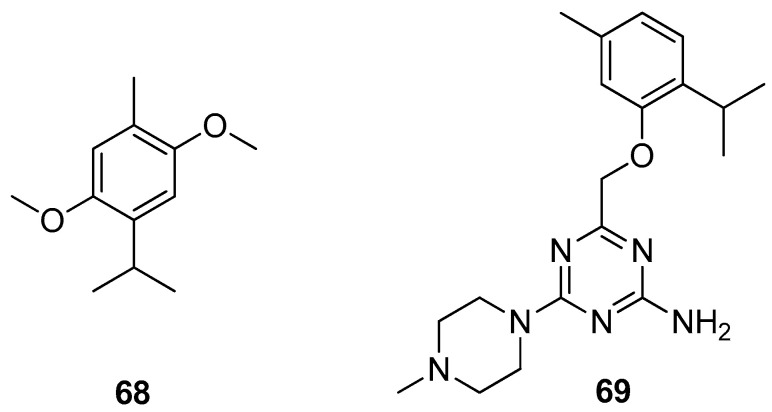
Thymol derivatives with pharmacological activity.

Haddad et al. isolated thymohydroquinone dimethyl ether (THQ) **68** from the sample of *Anaphalis triplinervis* (Sims) Sims ex C.B.Clarke essential oil (EO) (Figure 15). The authors also investigated the antiviral activity of *Anaphalis triplinervis* (Sims) Sims ex C.B.Clarke EO and its major compound THQ against ZIKV. Virological assays were performed on human lung epithelial A549 cells infected with either GFP reporter ZIKV or epidemic viral strain. To evaluate the acute toxicity of THQ in vivo the zebrafish assay was used. The results demonstrated that both EO and THQ inhibit ZIKV infection in human cells with IC_50_ values of 38 and 45 μM. It was noted that the inoculation of THQ at the antiviral effective concentration does not lead to any stress and does not impact fish survival [54].

Many studies have demonstrated that serotonin receptors, especially the 5-HT6 subtype, indicate great suitability for the potential therapy of the topic civilization CNS diseases.

Latacz et al. performed an exploration into the group of 1,3,5-triazine-methylpiperazines and screened them for pro-cognitive action and anxiolytic-like effect in the behavioral in vivo and in vitro tests. The best results were observed for the thymol triazine derivative. Compound 4-((2-isopropyl-5-methylphenoxy)methyl)-6-(4-methylpiperazin-1-yl)-1,3,5-triazin-2-amine **69** (Figure 15) shows the most promising pharmacological and drug-likeness profile and seems to be of higher importance due to its structure lacking the indole and sulfone fragments and the best 5-HT6R binding properties [55].

## 4. Monoterpene Bicyclic Derivatives

### 4.1. Myrtenal

Myrtenal is a monoterpene present in many medicinal plants such as cumin, pepper, mint, and eucalyptus. Monoterpenes consist of two isoprene units. They naturally occur in plants and essential oils. They are antioxidant, anticancer, antidiabetic, and act as cyclooxygenase-inhibitors and immunostimulants [4,56,57,58,59,60]. 

**Figure 16:**
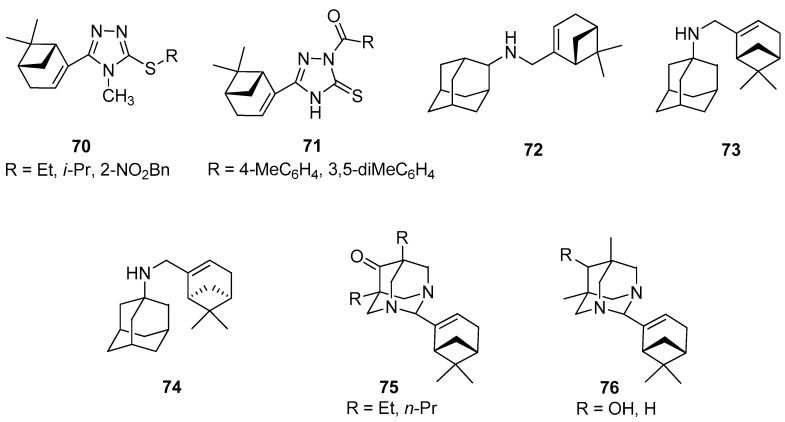
Selected myrtenal’s derivatives with significant antifungal, herbicidal, antiviral, anticancer, and analgesic activities.

1,2,4-Triazole derivatives are antimicrobial, antifungal, antitumor, anti-inflammatory, antituberculosis, and herbicidal properties [61,62,63]. A series of novel myrtenal-based 4-methyl-1,2,4-triazole-thioethers were synthesized by Duan and co-workers and tested as antifungal agents [64]. Antifungal activities of these compounds were evaluated by the in vitro method against Fusarium wilt on cucumber (*Fusarium oxysporum* f. sp. *cucumerinum*), apple root spot (*Physalospora piricola*), tomato early blight (*Alternaria solani*), speckle on peanut (*Cercospora arachidicola*), and wheat scab (*Gibberella zeae*) at 50 μg/mL. It was found that myrtenal-based compounds **70** such as 4-methyl-1,2,4-triazole-*i*-propylthioether **70b**, 4-methyl-1,2,4-triazole-*o*-nitrobenzylthioether **70c**, and 4-methyl-1,2,4-triazole-ethylthioether **70a** (Figure 16) exhibit excellent antifungal activity against *P. piricola* with inhibition rates of 98.2%, 96.4%, and 90.7%, respectively, showing better or comparable antifungal activity than that of the commercial fungicide azoxystrobin with an inhibition rate of 96.0%, which served as a positive control. Most of the target compounds have more enhanced activities than myrtenal, indicating that the incorporation of 1,2,4-triazole-thioether moiety into the myrtenal molecule was favorable to the increase of antifungal activity.

Duan’s group also focused their attention on the preparation of novel myrtenal-based 2-acyl-1,2,4-triazole-3-thiones [65]. These compounds were evaluated in vitro for their antifungal and herbicidal activities. Some of those myrtenal derivatives exhibit favorable antifungal activity at 50 mg/L. Myrtenal-derived 2-(*p*-methylbenzoyl)-1,2,4-triazole-3-thione **71a** demonstrates 83.7% and 72.5% inhibition rates against corn southern leaf blight (*Bipolaris maydis*) and apple root spot (*Physalospora piricola*), respectively, and myrtenal-derived 2-(3′,5′-dimethylbenzoyl)-1,2,4-triazole-3-thione **71b** has a 72.5% inhibition rate against *P. piricola* (the positive control commercial fungicide chlorothalonil with growth inhibition rates of 90.4% and 75.0% against *B. maydis* and *P. piricola*, respectively). Furthermore, most of the derivatives exhibit excellent herbicidal activity against the root-growth of rape (*Brassica campestris*) at 100 mg/L, with inhibition rates of 80.2% to 99.1%, showing much better herbicidal activity than that the commercial herbicide flumioxazin (positive control) with an inhibition rate of 63.0% (Figure 16). 

Adamantane derivatives have been widely used in clinical practice [66]. The applicability of compounds with both the adamantane and myrtenal moieties was investigated in various biological tests.

Suslov and co-workers synthesized a few new nitrogen-containing compounds with adamantane and myrtenal framework and studied the anxiolytic activity of the obtained products in male Balb/C mice in the elevated plus-maze (EPM) test. The results of this investigation showed that *N*-{[(1*R*,5*S*)-6,6-dimethylbicyclo[3.1.1]hept-2-en-2-yl]-methyl}adamantan-2-amine **72** exhibits tranquilizing activity and pronounces anxiolytic activity in the EPM test (Figure 16) [67]. Compound **72** was tested in vitro for antiviral activity against the influenza virus A/California/07/09 (H1N1)pdm09. The results indicated that the introduction of a myrtenal fragment led to an increase in the antiviral activity of adamantylamine derivatives against the adamantylamine resistant virus: The selectivity index of the majority of the synthesized amines was higher than that of rimantadine and amantadine [68]. Compound **73** was synthesized from 1-aminoadamantane and (–)-myrtenal and its cytotoxic activity against human cancer cells CEM-13, MT-4, and U-937 was studied. Adamantylamine **73** revealed a high activity against all tumor lines used (CTD_50_ = 12–21 µM) along with low toxicity for MDCK cells (CTD_50_ = 1500 µM). Furthermore, no genotoxic effect on normal cells was observed [69], whereas myrtenal-derived amine **74** synthesized from 1-aminoadamantane and (+)-myrtenal inhibited anti-Tdp1 activity with IC_50_ values of 6.1μM (Figure 16) [70].

Adamantane derivatives containing N atoms at the bridging positions manifested analgesic activity in vivo tests. Suslov’s group prepared a series of new compounds linking diazaadamantane and monoterpene moieties such as substituted 5,7-dimethyl-1,3-diazaadamantan-6-ones, which were vastly potent analogs of analgesic. The results showed that analgesic activity was completely lost if the 5- and 7-alkyl substituents were lengthened by one or two CH_2_ groups in 5,7-dialkyl-1,3-diazaadamantan-6-ones substituted with monoterpenoids in the aminal position **75**. In addition, compound **76a** with a 6-OH instead of the carbonyl group was found to be active in the visceral pain test and it does not show analgesic activity for tactile pain, while the reduction in the ketone to methylene group in diazaadamantane **76b** with (–)-myrtenal in the aminal position of the heteroadamantane structure affected the loss of analgesic properties in both tests (Figure 16) [71].

**Figure 17:**
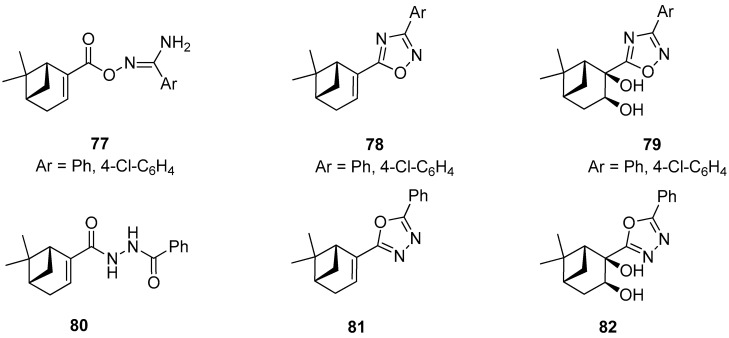
Monoterpene-based 1,2,4- and 1,3,4-oxadiazole derivatives.

Szakonyi and co-workers reported on the synthesis of (–)-myrtenal-based 1,2,4- and 1,3,4-oxadiazole derivatives. All prepared compounds along with their amidoxime type intermediates were screened in vitro for their antiproliferative activities against four human malignant cell lines using the MTT [3-(4,5-dimethylthiazol-2-yl)-2,5-diphenyltetrazolium bromide] assay. *O*-Acylated amidoxime **77** was found to have highly potent growth inhibitory activities with calculated IC_50_ values comparable to those of reference agent cisplatin. However, this molecule exhibited lower antiproliferative activities against the triple-negative breast cancer cell line (MDA-MB-231) than other cell lines used in gynecology. Unfortunately, the other compounds **78**–**82** exhibited a visibly weaker action against the ovarian cancer cell line (A2780) (Figure 17) [72].

### 4.2. Pinene

**Figure 18:**
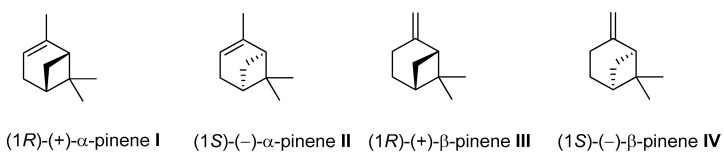
Structural formulas of *α*-pinene and *β*-pinene enantiomers.

Pinene (C_10_H_16_) is a bicyclic, double-bond terpenoid hydrocarbon, representing the family of monoterpenes. The *α*-pinene and *β*-pinene are two structural isomers found in nature, e.g., in pine (coniferous trees) essential oils (EO). Both isomers have two enantiomers (+) and (–) (Figure 18) and exhibit diverse pharmacological activities, e.g., antimicrobial, antiviral, antispasmodic, antifungal, antiviral, anticancer, antimalarial, antioxidant, and anti-inflammatory effects [73,74,75,76].

**Figure 19:**
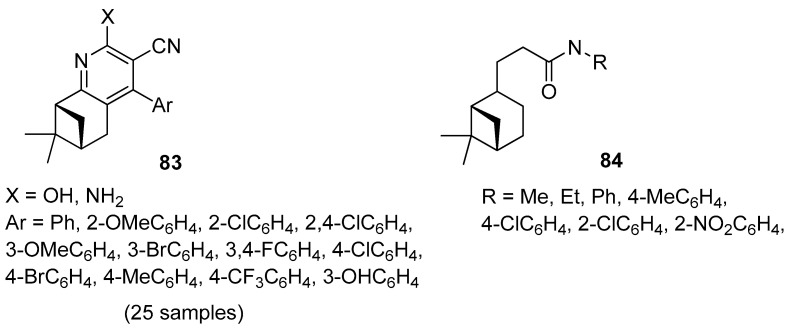
(–)-*β*-Pinene derivatives with antimicrobial and insecticidal activities.

Song and co-workers focused their attention on the preparation of novel 3-cyanopyridine derivatives of (–)-*β*-pinene 83 (Figure 19) [77]. All compounds were evaluated in vitro against four bacteria (*Klebsiella pneumoniae*, *Enterobacter aerogenes*, *Staphylococcus aureus*, *Staphylococcus epidermidis*) and a fungus (*Candida albicans*). The tested compounds exhibited moderate antibacterial activity against the Gram-positive bacteria *S. aureus*, *S. epidermidis*, weak antibacterial activity against the Gram-negative bacteria *K. pneumoniae*, *E. aerogenes*, and moderate antifungal activity against *C. albicans*. 4-(3,4-Difluorophenyl)-2-hydroxy-7,7-dimethyl-5,6,7,8-tetrahydro-6,8-methanoquinoline-3-carbonitrile **83h** with double fluoro substituents at *m*- and *p*-position on the pyridine ring demonstrates the best antimicrobial activity against overall strains.

Wang’s group synthesized a series of novel hydronopylformamides derivatives **84** from (–)-*β*-pinene and evaluated the repellency of these derivatives against the cockroach *Blattella germanica* (Figure 19). The results showed that only the compound with methyl, ethyl, phenyl, and 4-chlorophenyl units exhibited repellency against *B. germanica*. Compound 3-((1*S*,5*S*)-6,6-dimethylbicyclo[3.1.1]heptan-2-yl)-*N*-methylpropanamide **84a** shows a much better repellency than other derivatives and the traditional insect repellent *N*,*N*-diethyl-3-methylbenzamide (DEET) [78].

**Figure 20:**
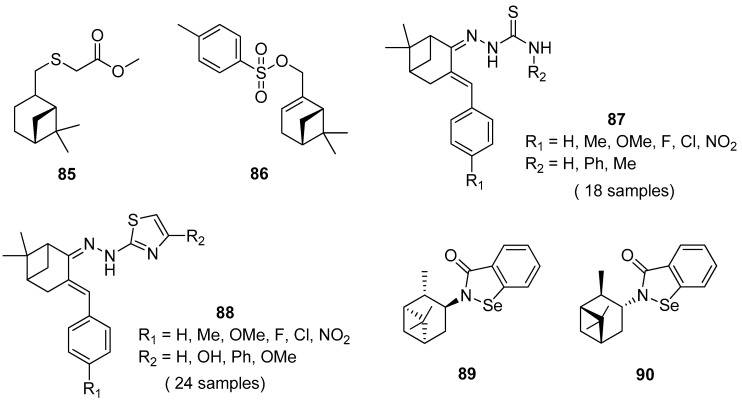
Representative pinene derivatives with pharmacological activity.

Thio derivatives of pinane series exhibit high antioxidant, antifungal, antiaggregation, anticoagulant, and pronounced antimycotic activity. Nikitina et al. [79] obtained pinane sulfide **85** by the addition of methyl hydrosulfanylacetate to the double bond of *β*-pinene and investigated this compound for hemocoagulation activity (Figure 20). The results indicated that pinane sulfide **85** (methyl [(1*R*,2*R*,5*R*)-(6,6-dimethylbicyclo[3.1.1]hept-2-yl)-methylthio]ethanoate) inhibits platelet receptor activity. It inhibits completely platelet aggregation induced by adrenaline, ADP, collagen, arachidonic acid, and also reduces the effect of ristomycin. This compound inhibits both platelet and coagulation hemostasis, which allows one to use it for the creation of promising antiaggregation drugs and the stabilization of blood products.

Ye et al. [80] designed and synthesized novel *α*-pinene derivatives as a potential antitumor agent. It was found that compound **86** (6,6-dimethylbicyclo[3.1.1]hept-2-en-2-yl)methyl-4-methylbenzenesulfonate) (Figure 20) exhibits the strongest inhibition on hepatoma carcinoma cell BEL-7402. The results showed that **86** demonstrates good antiliver cancer activity with the IC_50_ of 84.7 μM in vitro, and inhibits tumor growth in vivo with dose-dependent. The authors suggested that compounds **86**, apart from arresting the growth of hepatoma cells in the S phase, induce apoptosis in hepatoma cells, down-regulate the expression of C-myc, CDK2 and CyclinE, and up-regulate the level of p53.

Thiosemicarbazones, containing thiourea moiety, show significant biological—particularly anticancer—activities. Wang and co-workers [81] designed and synthesized a collection of nopinone-based thiosemicarbazone derivatives **87** as potent antineoplastic agents (Figure 20). Most of those thiosemicarbazones demonstrate considerable cytotoxic activity against three human cancer cell lines (MDA-MB-231, SMMC-7721, and Hela). Among thiosemicarbazone derivatives, the compound with phenyl group substituted at the amino position (R_2_ = Ph) and *p*-electron-donating group (R_1_ = OMe) exhibits the most potent antiproliferative activity against the tested cancer cell lines with the IC_50_ values of 2.79 ± 0.38, 2.64 ± 0.17 and 3.64 ± 0.13 μM, respectively, and low cytotoxicity on human normal embryo lung fibroblasts (Hlf-1).

Another example presents a library of *β*-pinene-based thiazole derivatives **88** (Figure 20) [82]. The obtained compounds were evaluated for their antitumor in vitro activities, and the results demonstrated that most target compounds showed potent antiproliferative activities against three human cancer cell lines. Especially, compound 4-(2-(2-(6,6-dimethyl-3-(4-nitrobenzylidene)bicyclo[3.1.1] heptan-2-ylidene)hydrazinyl)thiazol-4-yl)phenol (R_1_ = NO_2_, R_2_ = OH) displays excellent cytotoxic activity against Hela, CT-26, and SMMC-7721 cell lines with IC_50_ values of 3.48, 8.84, and 6.69 μM, respectively. These findings indicated that thiazole with a hydroxyl group attached to the phenyl group is favorable for increasing anticancer activity.

A series of new chiral *N*-terpenyl benzisoselenazol-3(2*H*)-ones with three monoterpene moieties—*p*-menthane, pinane, and carane—was synthesized by Ścianowski and co-workers [83]. The compounds were tested as antioxidants and anticancer agents. The best antioxidant activity and the highest antiproliferative potential were observed for *α*- and *β*-pinane *N*-substituted benzisoselenazolones **89** and **90** (Figure 20). The *N*-isopinocampheyl-1,2-benzisoselenazol-3(2*H*)-one **89** is the best peroxide scavenger and antiproliferative agent on the human promyelocytic leukemia cell line HL-60 with IC_50_ of 7.1 μM (HL-60 cell line).

**Figure 21:**
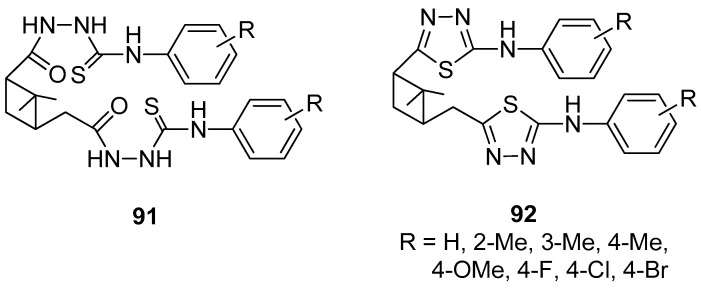
*α*-Pinene-based dithiadiazole compounds.

Lin et al. [84] presented the efficient synthesis of *α*-pinene-based dithiadiazoles **92** by the cyclization reaction of *α*-pinene-based disubstituted phenyl acyl amino thioureas **91**. The experiments showed that both compounds have weak herbicidal activity and the inhibition rates against fungi (*Brassica campestris* L., *Physalospora piricola*) are moderate except for 2,2′-methyl-substituted derivatives. A better plant growth-regulating activity was observed for derivatives with halo groups as compared to the other functional groups (Figure 21).

### 4.3. Camphor

Camphor, 1,7,7-trimethylbicyclo[2.2.1]heptan-2-one, is an abundant monoterpenoid with a bicyclic framework structure. This inexpensive natural compound occurs in dextrorotatory form, i.e., *R*,*R*-(+)-camphor, and was one of the first plant metabolites isolated in the chemically pure form [85]. Nowadays, it is obtained through distillation of camphor laurel tree wood (*Cinnamomum camphora* (L.) J.Presl) or otherwise by chemical transformation of other natural products, among other turpentines [86]. The laevorotatory form (*S*,*S*-camphor) only exists in a synthetic form or very small quantities in specific species of plants [87]. Camphor has been used widely as a fragrance in cosmetics and perfumes, a food flavorant, and in household cleaners [86]. This bicyclic compound possesses many biological activities: Insecticidal, analgesic, antimicrobial, antiviral, anticoccidial, antinociceptive, anticancer, and antitussive [86,88]. Importantly, (+)-camphor is one of the most commercially important aroma chemicals with an annual market value of US$80–100 million [89].

**Figure 22:**
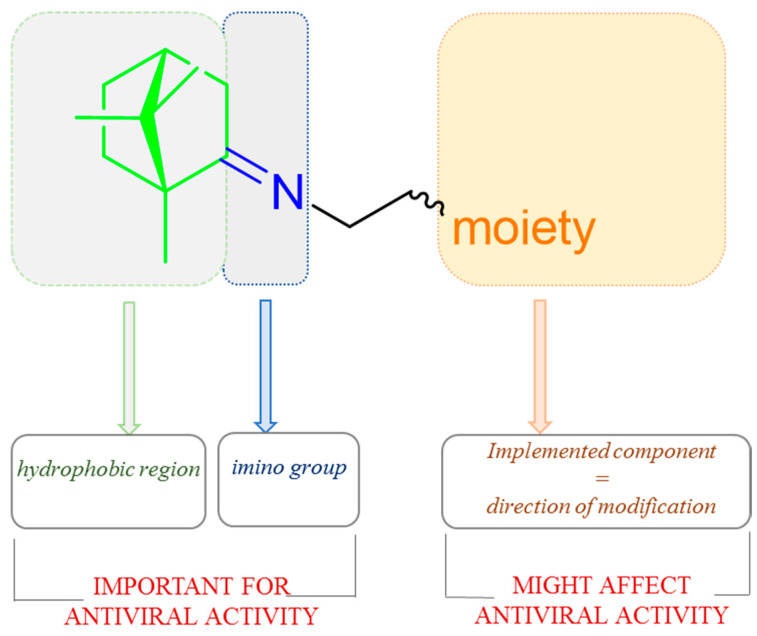
The design strategy for (+)-camphor-based imines with effectiveness biological activity.

Imines based on natural (+)-camphor proved to be a promising source of antimicrobial and antiviral agents [90]. Yarovaya et al. showed the designing strategy of (+)-camphor derivatives with the imine group (Figure 22) was prepared based on significant structural data described by the authors and shows the elements of the molecule which affects biological activity [91].

**Figure 23:**
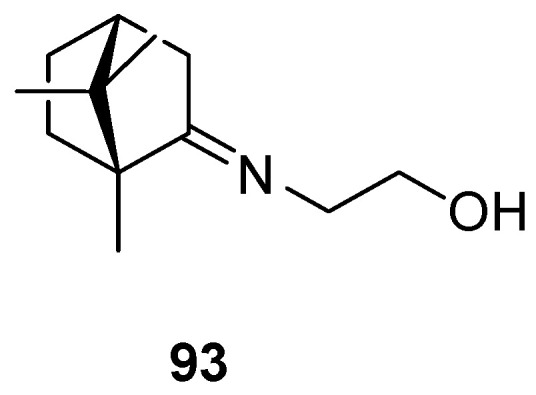
Camphecene—compound based on (+)-camphor and aminoethanol.

The most common (+)-camphor-based imine is camphecene **93** (Figure 23), 2-(*E*)-((1*R*,4*R*)-1,7,7-trimethylbicyclo[2.2.1]heptan-2-ylidene-aminoethanol, which was established as an effective inhibitor of influenza virus H1N1 with a selectivity index (SI) value of 500.3 [88] together with low toxicity. Camphecene remains promising as a potential antiviral due to the low pathogenicity of resistant viruses that may arise [92].

**Figure 24:**
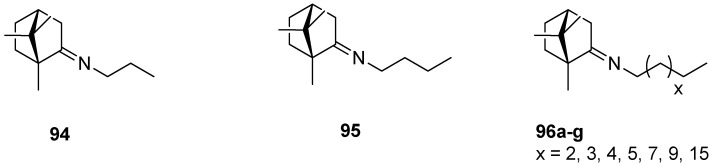
Aliphatic (+)-camphor imines with various lengths of the aliphatic chain.

An interesting series of works on the topic of (+)-camphor-based imine derivatives is presented by Sokolova et al. [88,93,94]. The authors pointed up a very efficient antiviral activity of synthesized monoterpene derivatives. In their 2017 work [94], designed compounds were prepared from commercially available natural (+)-camphor and various amines (Figure 24), whereas the procedures for the monoterpene derivatives were simple and reproducible. The prepared compounds were tested in vitro for antiviral activity. Most of those (+)-camphor-based imine derivatives—especially **94** and **95**—were capable of inhibiting the drug-resistant strains of influenza A virus subtypes A/Puerto Rico/8/34 and A/California/07/09 of H1N1pdm09. Analysis of the structure–activity relationship showed that the virus-inhibiting properties are strongly dependent on the length of an imine-conjugated moiety: Compounds with a shorter chain display higher activity, whereas the suppressing action of those aliphatic (+)-camphor imines with long aliphatic chains is lower.

**Figure 25:**
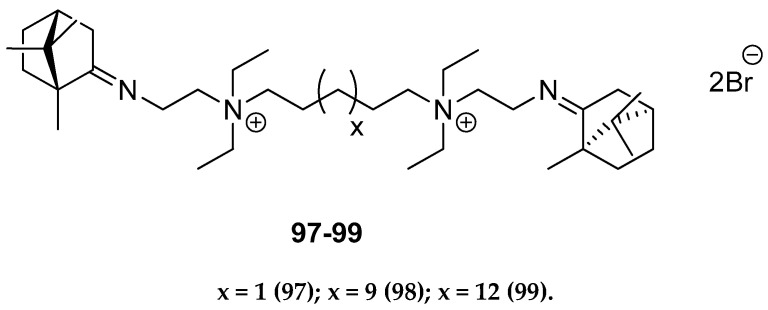
Quaternary ammonium (+)-camphor derivatives.

Other (+)-camphor derivatives, having two bicyclic monoterpene elements, two imine groups, and additionally two quaternary atoms in their structures connected with various lengths of the alkyl chain (Figure 25), were synthesized and tested through their biological applications [93]. Compounds with the pentyl **97** and nonyl **98** aliphatic chains are very efficient in inhibiting the influenza virus A(H1N1)pdm09 reproduction. Bis-quaternary ammonium bromide with a dodecyl alkyl chain **99** has the highest activity for the CEM-13 cells. The results suggest that tested dimeric bis-quaternary ammonium salts **97**–**99** might be used as components of drugs for suppressing malignant tumor development.

**Figure 26:**
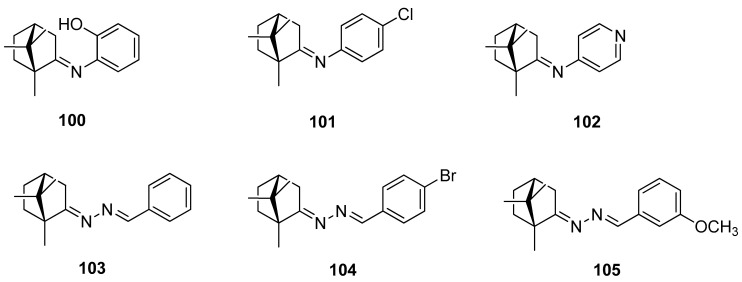
(+)-Camphor imine and hydrazone derivatives.

The next example, showing the use of naturally occurring camphor as a substrate, led to obtaining imines **100**–**102**, and also hydrazones **103**–**105**, which belong to *R*,*R*-(+)-camphor derivatives [95]. Figure 26 presents a few selected examples from a large set of synthesized compounds. Antimycobacterial activity against *Mycobacterium tuberculosis* ATCC 27294 was investigated for all the obtained (+)-camphor derivatives and the results were satisfactory. (*E*)-2-Hydroxy-*N*-(1,7,7-trimethylbicyclo[2.2.1]heptan-2-ylidene)aniline **100**, one of the imine derivatives, exhibits the best activity (minimal inhibitory concentration MIC = 3.12 µM). The antimycobacterial activity against *M. tuberculosis* of this (+)-camphor derivative is comparable to that of the antitubercular drug ethambutol. The other derivatives exhibit modest antimycobacterial activities at a range of 25–50 µM. In vitro tests against cancer cell lines show noncytotoxic activities for all the (+)-camphor derivatives.

**Figure 27:**
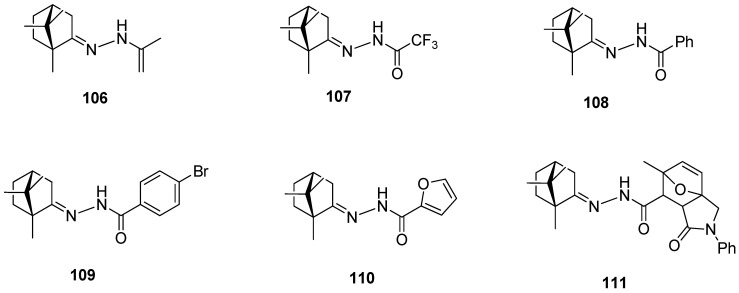
(+)-Camphor *N*-acylhydrazones with various aliphatic, aromatic, and heterocyclic substituents.

Another example presents a series of *N*-acylhydrazones containing a monoterpene fragment and different aliphatic, aromatic, and heterocyclic pharmacophore scaffolds (Figure 27) [96]. (+)-Camphor-based *N*-acylhydrazones exhibit inhibitory activity against vaccinia and influenza viruses. Compounds **108**, **109**, and **111**, containing an aromatic substituent, demonstrate low toxicity and the best activity against vaccinia virus. Furthermore, compound **111**, having aromatic and heterocyclic moieties, as the only one among the tested series of compounds, shows moderate activity against the influenza virus.

**Figure 28:**
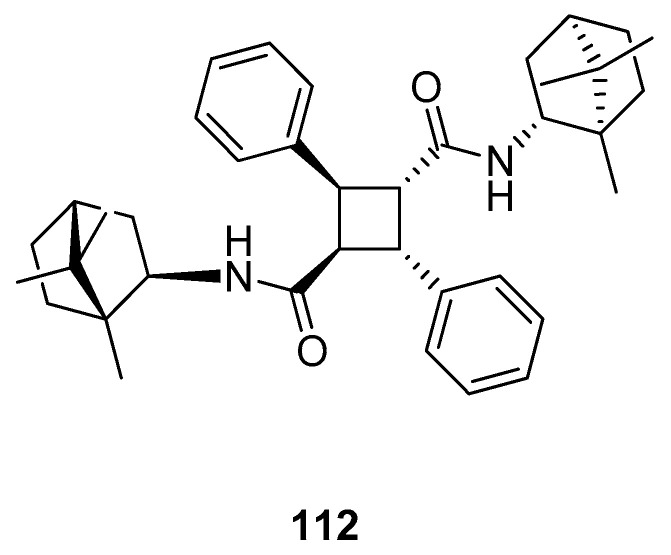
*α*-Truxillic acid derivative with (+)-camphor fragment.

Sokolova et al. [97] presented the efficient synthesis of *α*-truxillic acid derivatives containing a monoterpenoid fragment. The authors used natural terpene, (+)-camphor, as a starting monoterpenoid because of its high biological activity and commercial availability. Novel (+)-camphor derivative of *α*-truxillic acid, namely 2,4-diphenyl-*N*^1^,*N*^3^-bis((1*R*,4*R*)-1,7,7-trimethylbicyclo[2.2.1]heptan-2-yl)cyclobutane-1,3-dicarboxamide) **112** (Figure 28), was investigated for its analgesic activity in in vivo tests. The tested compound containing the cyclobutane unit and monoterpene bicyclic element at a dose of 10 mg/kg (per os) has good analgesic activity. Importantly, *α*-truxillic acid (tested for the same dose) did not show analgesic activity. Thus, the cause of considerable analgesic activity occurring in the tested compound is the introduction of (+)-camphor **112** fragment into *α*-truxillic acid.

**Figure 29:**
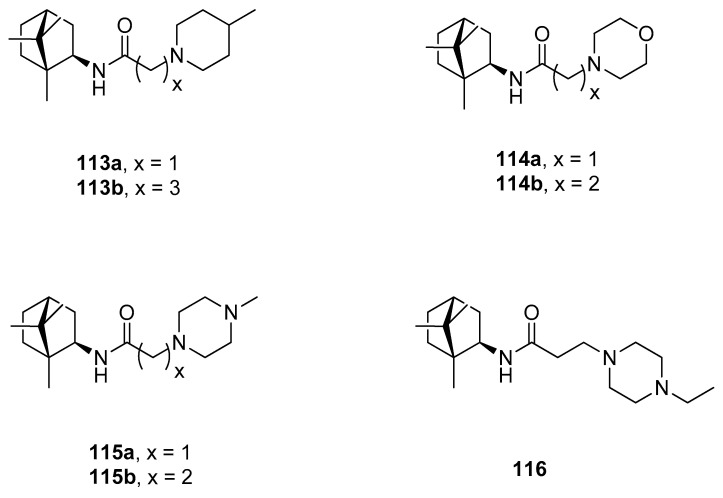
(+)-Camphor amide derivatives with the heterocyclic fragment.

Another example of the camphor amide derivatives is presented in Figure 29. Those heterocyclics were tested as potential vaccinia virus (VV) inhibitors [98]. Bioassay results revealed that synthesized amides with 4-methylpiperidine **113a** and morpholine **114b** fragments show the best inhibitory activity against VV with the IC_50_ values of 2.5 and 7.5 µM, respectively, with low cytotoxicity.

**Figure 30:**
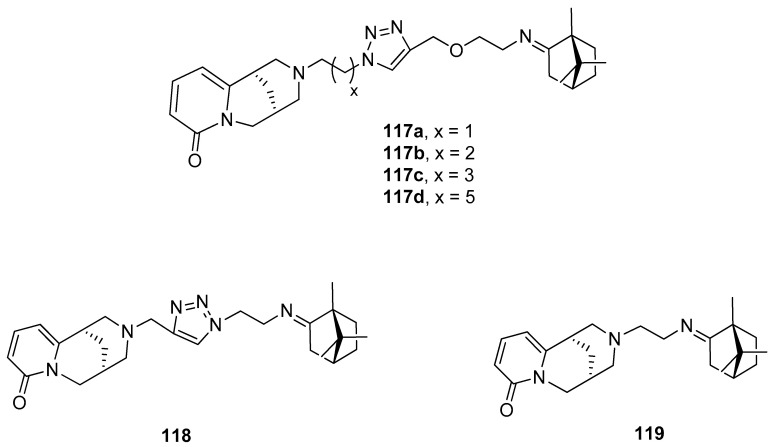
(+)-Camphor based (–)-cytisine with the heterocyclic fragment.

**Figure 31:**
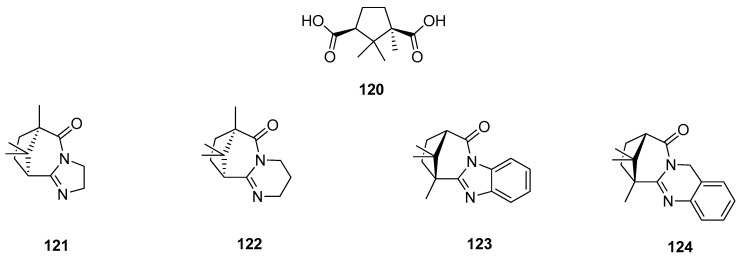
Polycyclic nitrogen-containing heterocyclic compounds (**121–124**) being (+)-camphoric acid (**120**) derivatives.

A series of hybrid molecules based on (+)-camphor derivatives and quinolizidine alkaloid (–)-cytisine (Figure 30) were presented by Artyushin et. al. [99] and tested for their cytotoxicity and virus-inhibiting activity. It was proven that the antiviral activity is affected by the length and nature of linkers between cytisine and (+)-camphor moieties. Compound **117d**, which contains the cytisine fragment separated from triazole ring by –C_6_H_12_– aliphatic linker shows the highest activity with relatively low toxicity (CC_50_ = 168 μmol, IC_50_ = 8 μmol, SI = 20). Its selectivity index appears higher than that of the commonly known reference compound: Rimantadine.

(+)-Camphoric acid **120** (Figure 31), which is the product of oxidation of (+)-camphor, was a substrate for an effective technique for the one-stage synthesis of new polycyclic nitrogen-containing compounds [100]. In this work, various aliphatic and aromatic diamines were selected as reagents for target polycyclic molecules because of the considerable role in the medicinal and organic chemistry of nitrogen-containing heterocyclic compounds. In vitro screening for the activity of the influenza virus A was performed for all synthesized polycyclic nitrogen-containing heterocyclic compounds, and synthetic analogs of natural alkaloids. Figure 31 shows selected structures (**121**–**124**), which were most effective in inhibiting the influenza virus A (H1N1). Compound **124**, exhibiting the best antivirus agent among others, has inhibitory activity against different strains of influenza virus A. This quinazoline like agent **124** has inhibitory activity against strain H5N2 comparable to reference compounds. In the case of effective inhibiting of strain H1N1, molecule **124** indicates an antivirus activity exceeding that of reference compounds.

**Figure 32:**
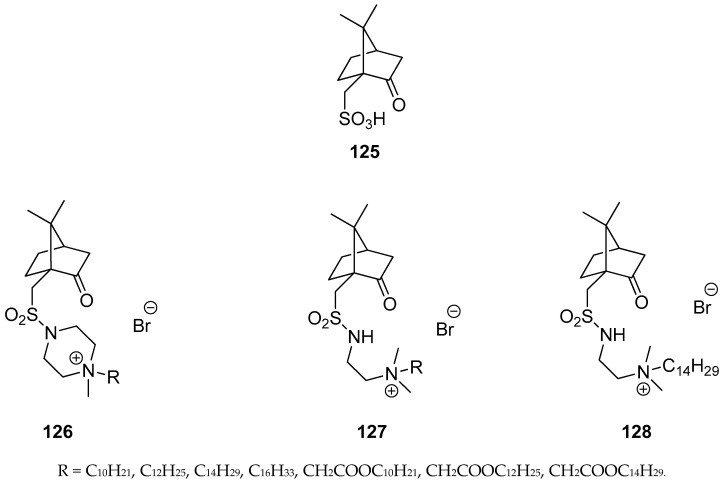
Quaternary ammonium salts (**126–128**) derived from (1*S*)-(+)-camphor-10-sulfonic acid (**125**).

Mikláš et al. presented an interesting set of homochiral quaternary ammonium sulfonamides bearing hydrophobic camphor derived moieties [101]. The authors used commercially available (1*S*)-(+)-camphor-10-sulfonic acid **125** (Figure 32), as a precursor for targeted monoterpene-based sulphonamides molecules **126**–**128** (Figure 32). Optically active quaternary ammonium bromides **126**–**128** containing camphor components were tested as antibacterial and antifungal agents and almost all of them show important antimicrobial activities. Compound *N*-{2-[((1*S*, 4*R*)-7,7-dimethyl-2-oxobicyclo[2.2.1]heptan-1-yl)methylsulfonamido]ethyl}-*N*,*N*-dimethyltetradecan-1-aminium bromide **128** is regarded as highly active against tested bacteria and fungi. The microbiological activity of this bromide **128** is approximately 25 times more effective against *S. aureus* and *C. albicans* and 100 times more active against *E. coli* than commercially used antimicrobial standard: Benzalkonium bromide.

**Figure 33:**
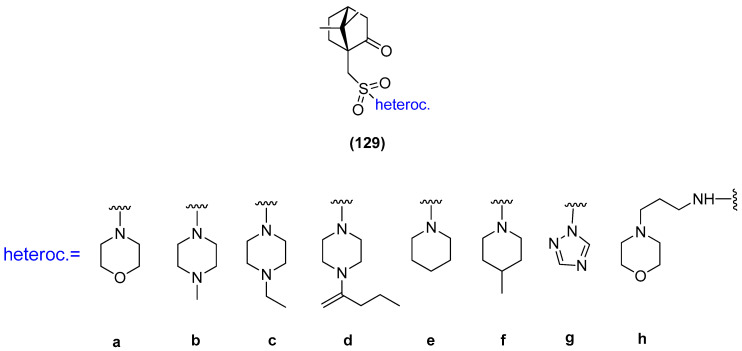
(1*S*)-(+)-Camphor-10-sulfonamide derivatives.

Another example, where the (1*S*)-(+)-camphor-10-sulfonic acid was used as a precursor of *N*-heterocycle compounds, is the work where compounds from the group of (1*S*)-(+)-camphor-10-sulfonamide derivatives **129** (Figure 33) were investigated as antiviral inhibitors against filoviruses [102]. The derivatives bearing morpholine **129a** and triazole **129g** moieties exhibit the highest inhibitory activity towards the Ebola virus glycoprotein, with the effectiveness level comparable to that of the reference drug. The excellent results presented in this paper allow us to predict that subsequent monoterpene bicyclic derivatives might be efficient inhibitors of especially dangerous viral infections.

### 4.4. Borneol

Borneol (1,7,7-trimethylbicyclo[2.2.1]heptan-2-ol) is bicyclic monoterpenoid alcohol that exists as two enantiomers in the D and L forms that occur in the essential oils of numerous medicinal plants, such as valerian (*Valeriana officinalis* subsp. *collina* (Wallr.) Nyman), chamomile (*Matricaria chamomilla* L. Rydb.), and lavender (*Lavandula officinalis* Chaix). It has four configurations that correspond to different positions of the hydroxyl group. Borneol is used—among others—in perfume and cosmetics manufacturing, agriculture and wood, and pharmaceutical industries. In China’s and India’s traditional medicine, borneol has long since been used as a remedy against gastrointestinal diseases. (–)-Borneol and its derivatives are antimicrobial [103], anti-inflammatory [104], and antiviral [105]. Borneol has been shown to promote drugs crossing through the blood–brain barrier, thus improving the efficiency of these drugs [106].

The particular biological properties of (–)-borneol derivatives belonging to the ester compounds significantly affect their applicability in medical areas, especially for diverse therapeutic fields [104,107,108,109]. (–)-Borneol esters are also found in a few plant families: For instance, the hydroxycinnamic variety in *Eupatorium deltoideum* Poepp. ex Spreng. and *Piper caninum* Blume. Such natural esters show a broad spectrum of biological properties. Bornyl caffeate and coumarate are highly antibacterial [110].

Bornyl salicylate—commonly known salicylic derivative, obtained by esterification of salicylic acid and (–)-borneol in the early 20th century—was used topically in inflammatory diseases. Interestingly, it was only in 2012 that the first detailed analysis of the anti-inflammatory effects of bornyl salicylate (BS) in experimental models of acute inflammation was done [104]. That research showed that signs of acute toxicity are not observed either in male or female mice. The authors revealed that the tested pharmaceutical compound is anti-inflammatory, which is related to the decrease in pro-inflammatory mediators because the treatment with bornyl salicylate is effective in the reduction of paw edema in both early and late phases.

**Figure 34:**
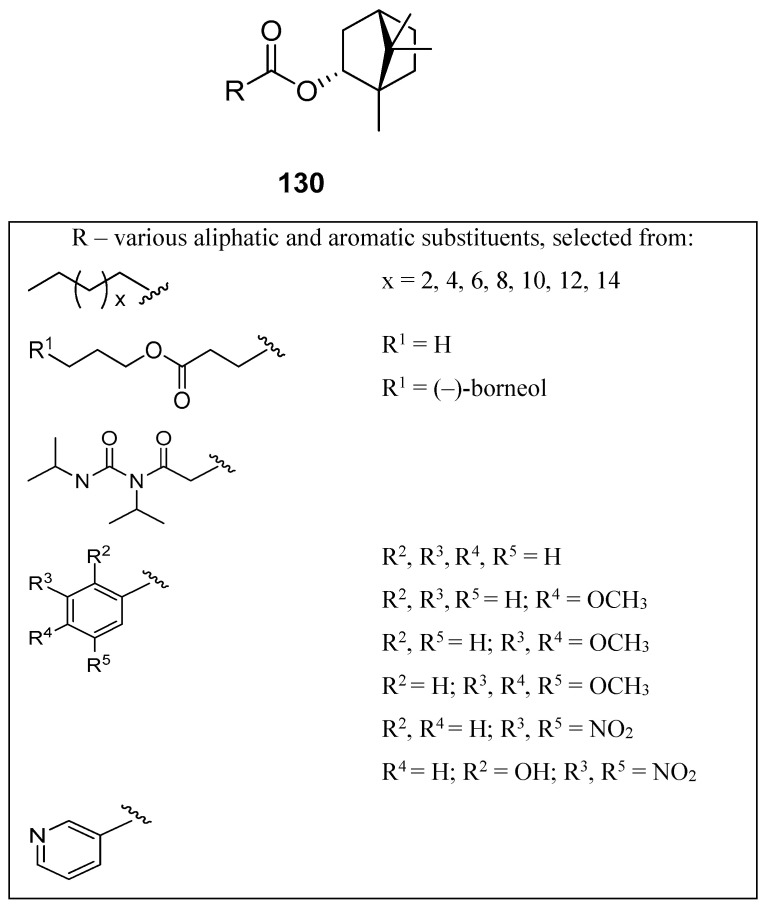

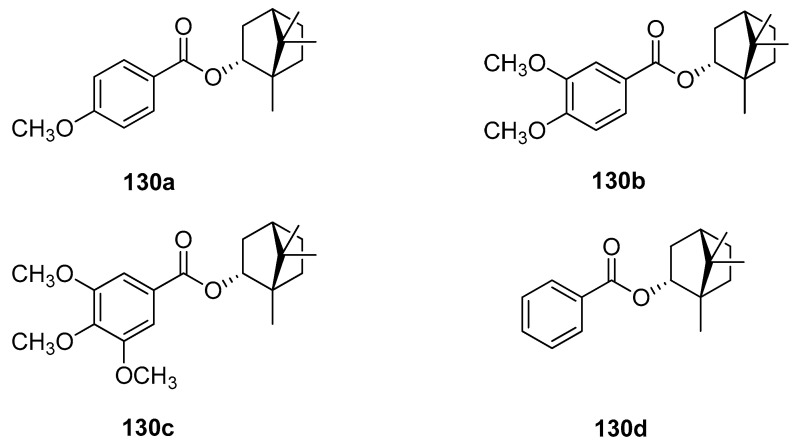
Aliphatic and aromatic esters of (–)-borneol derivatives were tested as antimicrobial agents [109] and as antiproliferative and antioedematogenic agents [108].

A large set of monoterpene derivatives that belong to the group of (–)-borneol esters were designed, synthesized, and examined with a view to their antimicrobial activity [109]. Desired compounds were prepared by the conventional and microwave-assisted methodology and the obtained (–)-borneol derivatives contained various aliphatic and aromatic moieties (Figure 34); all of them (**130**) were tested for their antibacterial and antifungal properties. Interestingly, among all examined monoterpene esters only those with methoxylated aromatic substituents (**130a**–**c**) show noteworthy microbiological results. The antimicrobial assays confirmed that compounds **130a**, **130b**, and **130c** exhibit promising antibacterial activity for some microorganisms, which is comparable to commonly used standard—ampicillin (MIC_50_ = 62.5 µM). Bornyl 3′,4′-dimethoxybenzoate **130b** shows high antimicrobial activity against all tested bacteria and fungus.

The presented set of aliphatic and aromatic esters of (–)-borneol derivatives (Figure 34) was also tested by the same research group for their antiproliferative and antioedematogenic properties [108]. Compounds **130c** and **130d** show a pronounced cytostatic activity against various tumor cell lines, which makes them useful models for the development of alternative drugs that might be effective and highly promising in the treatment of cancer. Additionally, compound **130d** [(1*S*,2*R*,4*S*)-1,7,7-trimethylbicyclo[2.2.1]heptan-2-yl benzoate] shows potential antioedematogenic activity being effective in the reduction of oedematogenic response in all evaluated periods. This aromatic ester of (–)-borneol derivative might be useful in the development of new antiinflammatory drugs.

Another research group [107] tested the in vitro effects of (1*S*,2*R*,4*S*)-1,7,7-trimethyl-bicyclo[2.2.1]heptan-2-yl-3′,4′,5′-trimethoxy benzoate **130c** and (1*S*,2*R*,4*S*)-1,7,7-trimethyl-bicyclo[2.2.1]heptan-2-yl benzoate **130d** on the growth and ultrastructure of *Trypanosoma cruzi*. These two (–)-borneol ester derivatives exerted an antiproliferative effect on the epimastigote forms of the parasite and might be used as a potential source for the development of more effective and safer chemotherapeutic agents against *T. cruzi* infections.

**Figure 35:**
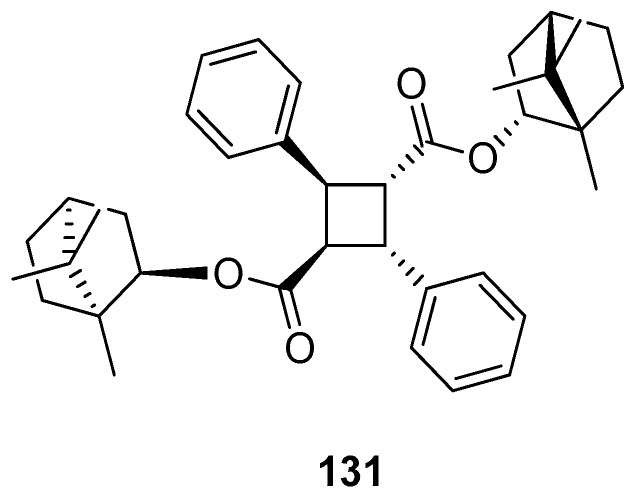
*α*-Truxillic acid derivative with (–)-borneol fragment.

Another work showing a new (–)-borneol ester derivative [97] focuses on the efficient synthesis of bis((1*R*,4*R*)-1,7,7-trimethylbicyclo[2.2.1]heptan-2-yl) 2,4-diphenylcyclobutane-1,3-dicarboxylate **131** using *α*-truxillic acid and monoterpenoid bicyclic (–)-borneol as the substrates (Figure 35). The new terpene compound obtained by Sokolova et al. exhibits a considerable analgesic activity. *α*-Truxillic acid (tested for the same dose) does not show analgesic activity, thus the monoterpenoid fragment is a structurally important element of the molecule, influencing its activity.

**Figure 36:**
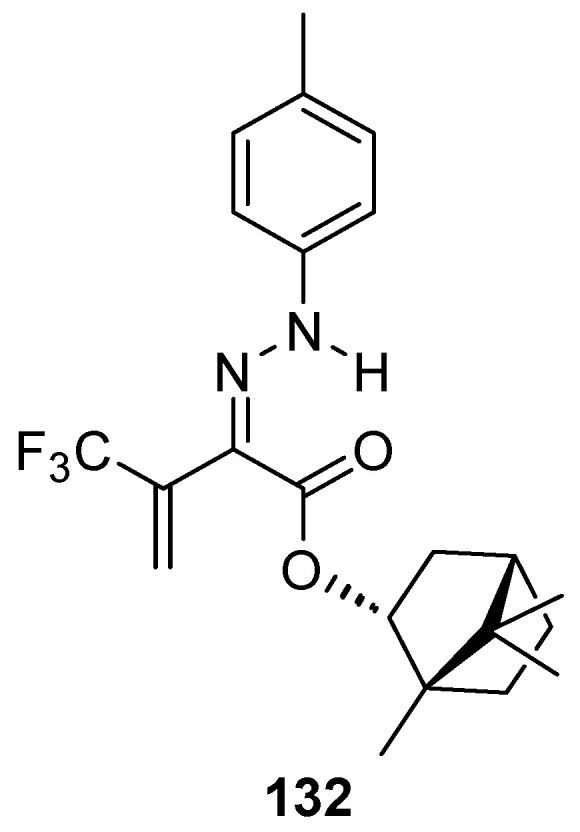
Ester derivative of natural occurring (–)-borneol with hydrazinylidene group.

Another (–)-borneol derivative with an ester group, namely bornyl (2*Z*)-4,4,4-trifluoro-2-[2-(4-methylphenyl)hydrazinylidene]-3-oxobutanoate **132** (Figure 36), was studied as an inhibitor of the main human isoenzymes involved in the biotransformation of ester-containing drugs: hCES1 and hCES2 [10]. The tested ester derivative of natural occurring (–)-borneol with the hydrazinylidene group is more active and selective against hCES1 than against hCES2. This compound inhibits hCES1 with the IC_50_ values of 0.098 ± 0.008 μM and has inhibitory activities against hCES2 with an IC_50_ value of 2.10 ± 0.18 μM.

**Figure 37:**
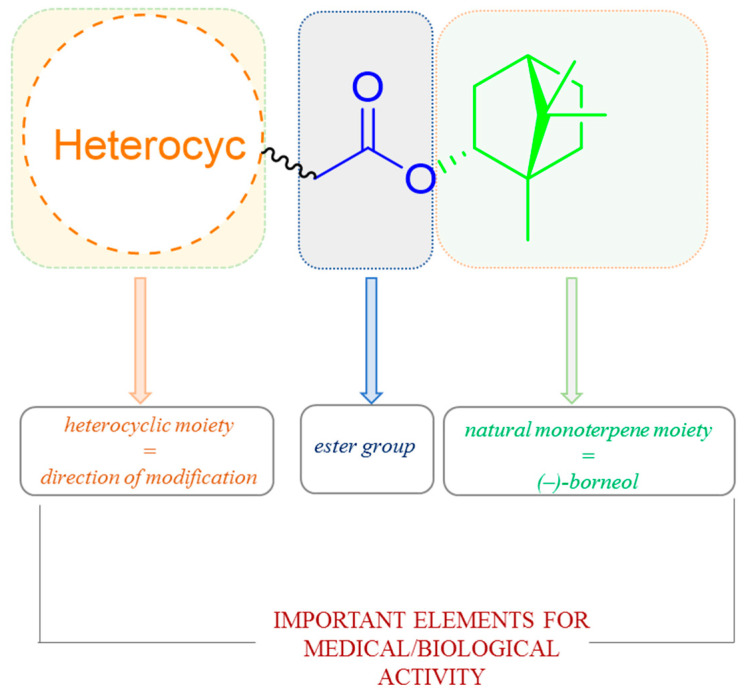
The design strategy for heterocyclic (–)-borneol-based esters with the effectiveness of the biological activity.

It is commonly known that heterocyclic compounds and their various derivatives due to their beneficial biological, pharmacological, and medical properties have attracted great attention from medicinal chemistry. Commonly mentioned examples are derivatives of benzimidazole, benzoxazole, and benzothiazole, which possess a wide range of biological effects, mainly antiulcer, anti-inflammatory, antiviral activity, antihypertensive, and analgesic [111,112,113,114]. On the other hand, the presence of 1,7,7-trimethylbicyclo[2.2.1]heptane scaffold of designed molecules induces many biological activities of prepared compounds, for instance high inhibitory activity of molecules on the replication of influenza A virus [88,94,95]. Hence, works where the compounds containing those two structural elements are broadly tested and significant biological activity are demonstrated. Importantly, most of such structural features of terpene-heterocyclic molecules also possess ester linker (Figure 37).

**Figure 38:**
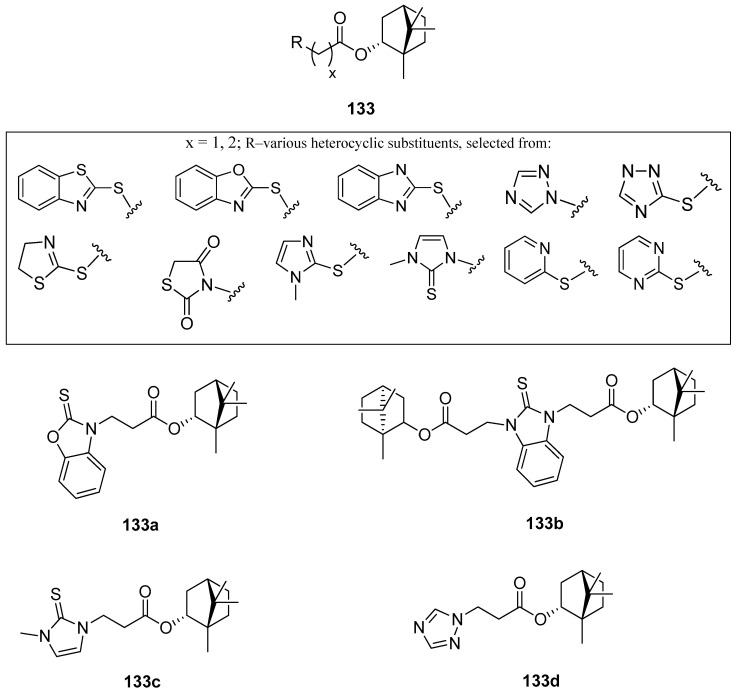
(–)-Borneol ester derivatives with different *N*- and *S*-nucleophiles.

One of the works on this subject is an article studying various heterocyclic (–)-borneol derivatives with benzothiazole, benzoxazole, benzimidazole, and other heterocycles moieties (Figure 38) [105]. Those compounds were analyzed due to their biological properties, including their possible use as antiviral, antiulcer, and analgesic agents. The inhibition of the influenza virus replication was evaluated with the A/Puerto Rico/8/34 (H1N1) virus, while rimantadine, amantadine, and deitiforin were tested as reference compounds, due to the structural similarity of their rigid cage fragments relative to heterocyclic (–)-borneol derivatives. Compound bearing two terpene moieties attached to 1*H*-benzimidazole-2-thiol core **133b** (Figure 38) exhibit considerable antiviral properties. Compounds with 1-methyl-1*H*-imidazole-2(3*H*)-thione **133c** and 1,2,4-triazole **133d** (Figure 38) fragments should be included as heterocyclic (–)-borneol derivatives exhibiting high antiviral activity. Compound **133a** containing benzoxazole-2-thiol fragment shows significant antiulcer activity. The examination of the gastric mucosa of the animals treated with **133a** (–)-borneol derivative prior to the indomethacin administration demonstrated a considerably reduced ulceration rate (PI 0.8) in comparison with the animals of the control group (PI 5.0). The results indicated that the antiulcer activity score of compound **133a** (AA with the value of 6.5) was higher than the same score for the reference compound (AA with a value of 3.8). While considering the analgesic activity of the presented (–)-borneol derivatives only compound **133d** tends to induce hyperalgesia, reducing the time of animal pain response. Some compounds of the large group of (1*S*,2*R*,4*S*)-1,7,7-trimethylbicyclo[2.2.1]-heptan-2-yl 2-chloroacetates and (1*S*,2*R*,4*S*)-1,7,7-trimethylbicyclo[2.2.1]heptan-2-yl 3-chloropropanoates combined with various *N*- and *S*-nucleophiles exhibit promising antiviral and antiulcer activity. Therefore, those heterocyclic (–)-borneol derivatives should be considered as important and highly encouraging compounds for further development of more potent inhibitors of influenza viruses and antiulcer agents.

**Figure 39:**
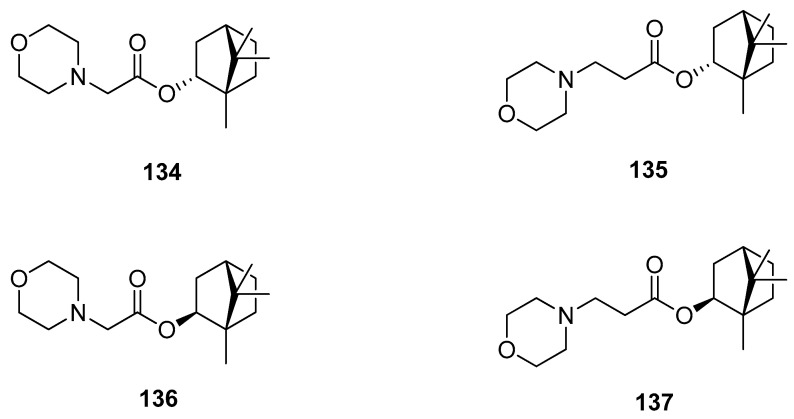
(–)-Borneol and (–)-isoborneol derivatives with morpholine fragment.

Another example presents various (–)-borneol and (–)-isoborneol (with different stereochemistries of the hydroxyl group) derivatives bearing a morpholine fragment as potential inhibitors of the influenza A virus (Figure 39) [115]. Compounds **134**, **135**, and **137** exhibit particularly high antiviral activity against the influenza virus A/Puerto Rico/8/34 (H1N1). The structure–activity analysis of those heterocyclic (–)-borneol derivatives revealed that a bulky lipophilic unit in the form of 1,7,7-trimethylbicyclo[2.2.1]heptan scaffold is required to obtain high results of antiviral activity.

**Figure 40:**
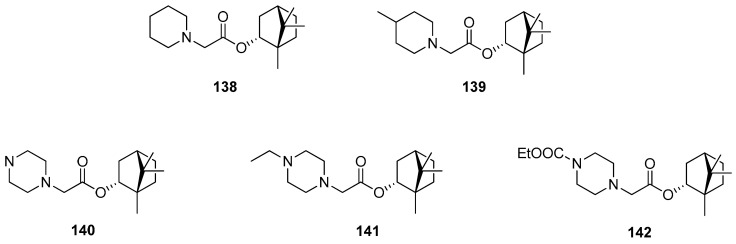
*N*-Heterocyclic esters of (–)-borneol derivatives as antiulcerogenic agents.

Borisova et al. presented the antiulcerogenic activity of heterocyclic (–)-borneol derivatives [116]. Some selected structures of the prepared set are shown in Figure 40. Compounds **140** and **142** containing piperazine moiety demonstrate antiulcerogenic activity on the indomethacin-induced ulcer model. Moreover, those (–)-bornyl ester derivatives possess a considerable antiulcerogenic effect, which is comparable to that shown by Omeprazole and Famotidine, used as standards. Bornyl 2-piperazinoacetate with a non-substituted piperazine moiety **140** shows maximum antiulcerogenic activity. Furthermore, this ester has a significant gastroprotective effect on the ethanol-induced ulcer model, which was similar to the one presented by Omeprazole. Therefore, further biological research into this (–)-borneol derivative containing piperazine moiety **140** should be aimed at the implementation of this compound as an antiulcerogenic agent.

**Figure 41:**
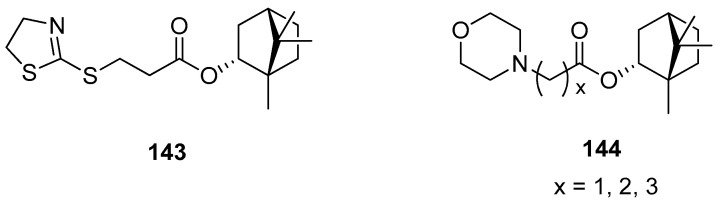
*N*-Heterocyclic esters of (–)-borneol derivatives as vaccinia virus inhibitors.

Another work presents (–)-bornyl ester derivatives with *N*-containing heterocycles as vaccinia virus (VV) inhibitors [98]. Some of the structures of the large set designed and synthesized by Sokolova et al. are shown in Figure 41. Bioassay results confirmed that four (–)-borneol derivatives (**143**, **144**) exhibit the best inhibitory activity against VV and low cytotoxicity. The authors emphasized two important structural issues that affect high anti-VV activity. Firstly, SAR studies suggest that the incorporation of the *N*-heterocyclic unit into the 1,7,7-trimethylbicyclo[2.2.1]heptane scaffold caused a significant improvement to antiviral activity. Secondly, (–)-borneol does not exhibit activity against VV. Thus, the presence of both heterocyclic elements and 1,7,7-trimethylbicyclo[2.2.1]heptane fragment is responsible for the antiviral activity against VV. The effectiveness of presented esters (Figure 41, compounds **143** and **144**) is comparable to the reference drug Cidofovir and suggests that those (–)-borneol heterocycle derivatives should be considered as potential anti-VV drug candidates.

Over 170 natural derivatives were screened in search of currently no approved antiviral therapy of Marburg virus disease (MVD) [117]. The (–)-bornyl ester derivatives containing saturated *N*-heterocycles exhibited the highest antiviral activity and can specifically inhibit MarV entry with the efficiency being comparable to that of the previously described verapamil. (–)-Borneol itself is not active against both tested pseudotypes. Among low-toxic (–)-borneol derivatives, some of the compounds inter alia **138**–**141** turned out to be relatively specific inhibitors of MarV-GP mediated infection (SC > 10). Compound **139** containing a methylpiperidine moiety exhibits the highest virus-specific activity, which is twice as high as that of the reference.

**Figure 42:**
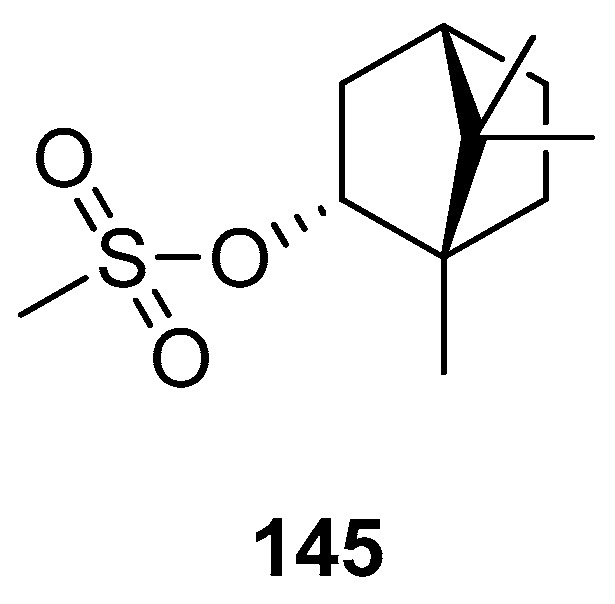
(–)-Borneol derivative, 1,7,7-trimethylbicyclo[2.2.1]hept-2-yl methane sulfonate as antibacterial agent.

Biological activities were also determined for another structural type of derivatives of (–)-borneol. One of the examples is 1,7,7-trimethylbicyclo[2.2.1]hept-2-yl methane sulfonate **145** (Figure 42), obtained from the reaction of (1*S*)-*endo*-(–)-borneol with methanesulfonyl chloride [118]. The antibacterial effect of compound **145** is comparable with that of the commonly used reference—chloramphenicol.

**Figure 43:**
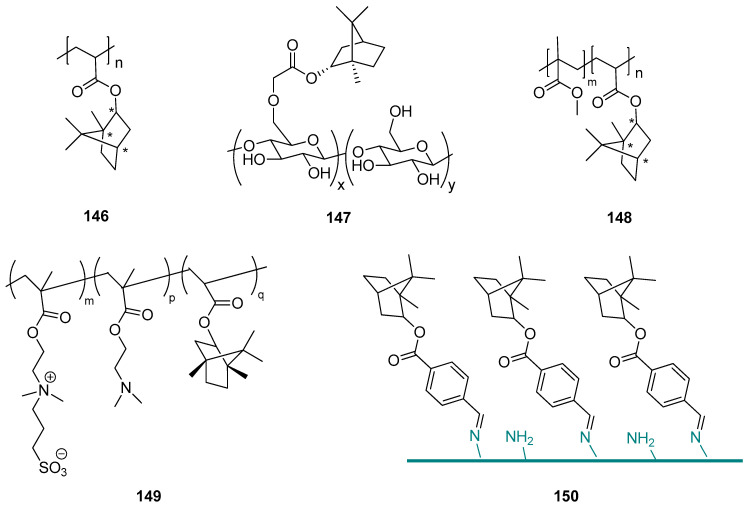
Borneol-based polymer.

Terpene compounds might also be used as substrates for the synthesis of specific polymer biomaterials as effective fouling resistant materials with lower cytotoxicity. An example of such an application is the utilization of natural borneol in designing a novel antibacterial material and developing an advanced strategy for antibacterial adhesion through polymer surface stereochemistry [119]. The authors used *endo*-L-borneol: (1*S*,2*R*,4*S*), *endo*-D-borneol: (1*R*,2*S*,4*R*), and *exo*-isoborneol: (1*S*,2*S*,4*S*) and (1*R*,2*R*,4*R*) to prepare a series of borneol-based polymer polyborneolacrylates (PBAs) (Figure 43, compounds **146**) that showed unique antibacterial adhesion properties and significantly reduced bacterial attachment and biofilm formation. The PBA polymers were evaluated as noncytotoxic and should be considered a great potential for many biomedical applications. Superior antibacterial adhesion properties result from the borneol isomers on the material surface. This proof-of-concept improves polymer surface stereochemistry is an advanced strategy for antimicrobial adhesion, and the use of natural monoterpenes for this purpose is highly advisable.

This concept is confirmed by the other work done by the Wang group [103], who presented (–)-borneol grafting onto cellulose employing chloroacetyl chloride as a covalent linking agent in a two-step reaction. The obtained borneol-grafted cellulose (BGC) (Figure 43, compound **147**) material exhibits remarkable performance in antifungal adhesion and fungal growth inhibition, which suggests that grafted borneol moieties crucially influence the tactile sensing of fungal cells and, subsequently, their selectivity in adhesion. The simple modification consisting of adding small borneol molecules onto cellulose resulted in a significant conversion of the interfacial antifungal property and also opened a new opportunity for developing a new generation of antimicrobial celluloses. More crucially, both borneol and cellulose are natural products, therefore it should be concluded that it is possible to develop an advanced natural strategy for exploiting cellulose’s potential use in antifungal adhesion and colonization. This environmentally friendly material might be applied in biomedicine and for sanitary purposes.

Sun et al. [120] described borneol-modified poly(methyl methacrylate) PMMA based on a facile and effective stereochemical strategy, generating an antibacterial copolymer named P(MMA-*co*-BA)—compound **148** (Figure 43). This copolymer, which effectively prevents bacterial adhesion and is environmentally benign, might be successfully applied for the prevention of bacterial colonization in biomedical devices. Its unique antibacterial character depends on the percentage of BA segments in the copolymer.

The borneol-based polymers are hydrophobic and noncytotoxic, which can significantly improve the cell adhesion ability [121,122]; on the other hand, borneol isomers on the material surface contribute to the surface bacterial resistance properties [119,120]. Due to those data, Meng et al. [123] proposed polymerization with borneol based monomer, assuming that the hydrophobicity of zwitterionic polymers might increase, which will promote their cell adhesion ability without compromising their bacterial resistance properties. For this purpose, they synthesized hydrophobized zwitterionic copolymer P-(SBMA-*co*-DMA-*co*-ISA) (PSDI) (compound **149**, Figure 43), containing borneol based segment isobornyl acrylate (ISA) and zwitterionic moiety SBMA was prepared by sulfonation of P(DMA-*co*-ISA) (PDI). Together with tannic acid (TA), PSDI colloidal particles (CPs) were deposited onto the Ti alloy surface by electrophoretic deposition to form zwitterionic-based coatings. Due to the synergistic effect between the SBMA and chiral ISA functional units, tested composite coatings can effectively inhibit the adhesion of Gram-negative (*E. coli*) and -positive bacteria (*S. aureus*). Furthermore, cells can adhere and grow on the surface of CP-TA composite coating as a result of the appropriate hydrophobicity provided by the chiral monoterpene moiety. Meng et al., in their work, presented a new strategy for preparing zwitterionic-based multifunctional coating. The proposed composite coatings have great potential for metal implant surface modification to prevent bacterial infections.

Another example showed the development of stereochemical antimicrobial strategy by grafting (–)-borneol 4-formylbenzoate to chitosan using a stable Schiff base bond (compound **150**, Figure 43) [124]. The authors of that project received borneol-modified chitosan (BMC) as a potentially novel antimicrobial material. The biological tests were performed using Gram-negative *E. coli*, Gram-positive *B. subtilis*, and *A. niger*. BMC exhibits good antibacterial adhesion properties against both Gram-positive and Gram-negative bacteria. The BMC materials also demonstrate excellent antifungal adhesion performance (even for up to 29 days). Hence, modified chitosan with monoterpene moiety should be considered a broad-spectrum antimicrobial material. It enhances the protective effects of CF concerning the potential attachment of pathogens and maintains the skin flora. Therefore, BMC could have applications as a novel biomaterial-based on the stereochemical antimicrobial strategy and be useful in many biomedical and sanitary applications.

Another concept for creating an interesting biomaterial based on the (–)-borneol component is graphene oxide-borneol (GOB) composite [125], which can be synthesized by esterification of (–)-borneol with thiomalic-acid-modified graphene oxide (GO) sheets, of which thiomalic acid is used as the linker molecule to increase the number of surface carboxyl groups. GOB exhibits antifungal adhesion and growth inhibition. It was proven that the carbon stereochemistry of the GOB was essential for the powerful antifungal performance while the covalent banding between GO and borneol molecules ensured its safe and long-term antifungal characteristics.

## 5. Conclusions

Terpenes make up the largest group of secondary metabolites (over 50,000 substances) and due to their naturally widespread occurrence, they have become central to research activities worldwide. It has been demonstrated that the use of the available and cheap monoterpenes as building blocks for designing and synthesizing new and effective agents to treat several diseases and some viral and bacterial infections is a promising and progressing direction in medicinal chemistry.

A crucial benefit from using described compounds is that their resource is almost inexhaustible, which has also ensured their wide application in industry. Moreover, the use of monoterpenoids to design drugs often reduces the toxicity of the resulting compound, which is an important factor when considering the possible utilization and manufacture of new compounds. It is widely known that chemical modification of natural substances with various components is an important method for obtaining new biologically active compounds. This feature is particularly visible in the monoterpene derivatives presented in this review. Their biological activities are often much superior to the parent substances (monoterpenes); furthermore, they often exceed the commonly used standards in specialized areas of research.

The increased strength of activity is observed by supplement functionalized moiety (e.g., heterocyclic unit introduced into thymol, camphor, or borneol derivatives) or special group, like an ester group in borneol derivatives, or an imino group in camphor derivatives. Nowadays, this is a promising route as this is of utmost importance to utilize natural substances for discovering new compounds and to search for practical uses for the newly obtained materials. As presented in this paper, monoterpenes exhibit a large variety of biological activities that can be exploited in various branches of medicine and pharmacy. This review highlights the use of the selected beneficial monoterpenes and their derivatives for the treatment of various diseases. It should be mentioned that alongside the biological activities presented in this paper, the substances play important roles in preventing and remediating other diseases such as those of the heart and diabetes. Obtaining new structures based on monoterpene compounds is a dynamically developing area, and one should expect new interesting biological applications, as well as modifications to structures that will improve current results in biological research.

## Abbreviations

AA	the antiulcer activity
AChE	acetylcholinesterase enzymes
A/Puerto Rico/8/34 (H1N1) virus	influenza A virus, subtype
BA	borneol acrylate
BGC	borneol-grafted cellulose
BMC	borneol-modified chitosan
BS	bornyl salicylate
BuChE	butyrylcholinesterase
CEM-13	the cells of T-cellular human leucosis
CES	inhibitory activity against porcine liver
CF	chitosan fabric
CNS	central nervous system
COX-2	cyclooxygenase-2
CPs	colloidal particles
DEET	*N*,*N*-diethyl-3-methylbenzamide
DMA	2-(dimethylamino)ethyl methacrylate
DPPH	2,2-diphenyl-1-picrylhydrazyl
EO	essential oil
EPM test	elevated plus maze test
ESBL	extended-spectrum beta-lactamases
fMLP/CB	formyl-l-methionyl-l-leucyl-l-phenylalanine/cytochalasin B
GO	graphene oxide
GOB	graphene oxide-borneol
H5N2	influenza A virus, subtype
hCA I and II	cytosolic carbonic anhydrase I and II isoforms
ISA	isobornyl acrylate
LPS	lipopolysaccharide
MARV	Marburg virus
MMA	methyl methacrylate
MRSA	methicyllin-resistant *S. aureus*
MTT	3-(4,5-dimethylthiazol-2-yl)-2,5-diphenyltetrazolium bromide
NO	nitric oxide
PBA	polyborneolacrylate
P-(SBMA-*co*-DMA-*co*-ISA) (PSDI)	poly(sulfobetaine methacrylate-*co*-2-(dimethylamino)ethyl methacrylate-*co*-isobornyl acrylate)
PDI P(DMA-*co*-ISA)	hydrophobized zwitterionic copolymer
PI	Paul’s index in testes of antiulcer activity
PMMA	poly(methyl methacrylate)
SAR	structure-activity relationship
SBMA	sulfobetaine
SRB	sulforhodamine B
TA	tannic acid
THQ	thymohydroquinone dimethyl ether
VV	vaccinia virus
ZIKV	Zika virus

## References

[1] Kozioł A., Stryjewska A., Librowski T., Sałat K., Gaweł M., Moniczewski A., Lochyński S. (2014). An overview of the pharmacological properties and potential applications of natural monoterpenes. Med. Chem..

[2] Wojtunik-Kulesza K.A., Kasprzak K., Oniszczuk T., Oniszczuk A. (2019). Natural monoterpenes: Much more than only a scent. Chem. Biodivers..

[3] Barreto R.S.S., Albuquerque-Júnior R.L.C., Araújo A.A.S., Almeida J.R.G.S., Santos M.R.V., Barreto A.S., DeSantana J.M., Siqueira-Lima P.S., Quintans J.S.S., Quintans-Júnior L.J. (2014). A systematic review of the wound-healing effects of monoterpenes and iridoid derivatives. Molecules.

[4] Dragomanova S., Tancheva L., Georgieva M. (2018). A review: Biological activity of myrtenal and some myrtenal-containing medicinal plant essential oils. Scr. Sci. Pharm..

[5] Marchese A., Orhan I.E., Daglia M., Barbieri R., Di Lorenzo A., Nabavi S.F., Gortzi O., Izadi M., Nabavi S.M. (2016). Antibacterial and antifungal activities of thymol: A brief review of the literature. Food Chem..

[6] Dheer J.D., Singh D., Kumar G., Karnatak M., Chandra S., Verma V.P., Shankar R. (2019). Thymol chemistry: A medicinal toolbox. Curr. Bioact. Compd..

[7] van Der Heijden R., Jacobs D.I., Snoeijer W., Hallard D., Verpoorte R. (2004). The Catharanthus alkaloids: Pharmacognosy and biotechnology. Curr. Med. Chem..

[8] Carnesecchi S., Bras-Goncalves R., Bradaia A., Zeisel M., Gosse F., Poupon M.F., Raul F. (2004). Geraniol, a component of plant essential oils, modulates DNA synthesis and potentiates 5-fluorouracil efficacy on human colon tumor xenografts. Cancer Lett..

[9] Lei Y., Fu P., Jun X., Cheng P. (2019). Pharmacological properties of geraniol—A review. Planta Med..

[10] Makhaeva G.F., Elkina N.A., Shchegolkov E.V., Boltneva N.P., Lushchekina S.V., Serebryakova O.G., Rudakova E.V., Kovaleva N.V., Radchenko E.V., Palyulin V.A. (2019). Synthesis, molecular docking, and biological evaluation of 3-oxo-2-tolylhydrazinylidene-4,4,4-trifluorobutanoates bearing higher and natural alcohol moieties as new selective carboxylesterase inhibitors. Bioorg. Chem..

[11] Chavez M.I., Soto M., Cimino F.A., Olea A.F., Espinoza L., Díaz K., Taborga L. (2018). In Vitro antifungal activity of new and known geranylated phenols against *Phytophthora cinnamomi* rands. Int. J. Mol. Sci..

[12] Takamura H., Ohashi T., Kikuchi T., Endo N., Fukuda Y., Kadota I. (2017). Late-stage divergent synthesis and antifouling activity of geraniol–butenolide hybrid molecules. Org. Biomol. Chem..

[13] Yamanaka T. (1976). Catalytic properties of metal sulfates supported on γ-Al_2_O_3_ in the liquid-phase isopropylation of *m*-cresol with propylene. Bull Chem. Soc. Jpn..

[14] Grabowska H., Miśta W., Trawczyński J., Wrzyszcz J., Zawadzki M. (2001). A method for obtaining thymol by gas phase catalytic alkylation of *m*-cresol over zinc aluminate spinel. Appl. Catal. A Gen..

[15] Amandi R., Hyde J.R., Ross S.K., Lotz T.J., Poliakoff M. (2005). Continuous reactions in supercritical fluids; a cleaner, more selective synthesis of thymol in supercritical CO_2_. Green Chem..

[16] Gill T.A., Li J., Doppler M., Scofield S.R. (2016). Thymol-based submicron emulsions exhibit antifungal activity against *Fusarium graminearum* and inhibit *Fusarium* head blight in wheat. J. Appl. Microbiol..

[17] de Lira Mota K.S., de Oliveira Pereira F., de Oliveira W.A., Lima I.O., de Oliveira Lima E. (2012). Antifungal activity of *Thymus vulgaris* L. essential oil and its constituent phytochemicals against *Rhizopus oryzae*: Interaction with ergosterol. Molecules.

[18] Wattanasatcha A., Rengpipat S., Wanichwecharungruang S. (2012). Thymol nanospheres as an effective antibacterial agent. Int. J. Pharm..

[19] Miladi H., Zmantar T., Kouidhi B., Chaabouni Y., Mahdouani K., Bakhrouf A., Chaieb K. (2017). Use of carvacrol, thymol, and eugenol for biofilm eradication and resistance modifying susceptibility of *Salmonella enterica* of serovar *Typhimurium* strains to nalidixic acid. Microb. Pathog..

[20] Veras H.N., Araruna M.K., Costa J.G., Coutinho H.D., Kerntopf M.R., Botelho M.A., Menezes I.R. (2013). Topical antiinflammatory activity of essential oil of *Lippia sidoides* cham: Possible mechanism of action. Phytother. Res..

[21] Botelho M.A., Barros G., Queiroz D.B., Carvalho C.F., Gouvea J., Patrus L., Bannet M., Patrus D., Rego A., Silva I. (2016). Nanotechnology in phytotherapy: Antiinflammatory effect of a nanostructured thymol gel from *Lippia sidoides* in acute periodontitis in rats. Phytother. Res..

[22] Riella K., Marinho R., Santos J., Pereira-Filho R., Cardoso J., Albuquerque-Junior R., Homazzi S. (2012). Anti-inflammatory and cicatrizing activities of thymol, a monoterpene of the essential oil from *Lippia gracilis*, in rodents. J. Ethnopharmacol..

[23] Kumar D., Rawat D.S. (2013). Synthesis and antioxidant activity of thymol and carvacrol based Schiff bases. Biorgan. Med. Chem..

[24] Deng L.-L., Taxipalati M., Que F., Zhang H. (2016). Physical characterization and antioxidant activity of thymol solubilized Tween 80 micelles. Sci. Rep..

[25] Kang S.H., Kim Y.S., Kim E.K., Hwang J.W., Jeong J.H., Dong X., Park P.J. (2016). Anticancer effect of thymol on AGS human gastric carcinoma cells. J. Microbiol. Biotech..

[26] Gao T., Zhou H., Zhou W., Hu L., Chen J., Shi Z. (2016). The fungicidal activity of thymol against *Fusarium graminearum* via inducing lipid peroxidation and disrupting ergosterol biosynthesis. Molecules.

[27] Zhao J., Li Y., Liu Q., Gao K. (2010). Antimicrobial activities of some thymol derivatives from the roots of *Inula hupehensis*. Food Chem..

[28] Mathela C.S., Singh K.K., Gupta V.K. (2010). Synthesis and in vitro antibacterial activity of thymol and carvacrol derivatives. Acta Pol. Pharm..

[29] Chauhan K.R., Le T.C., Chintakunta P.K., Lakshman D.K. (2017). Phyto-fungicides: Structure activity relationships of the thymol derivatives against *Rhizoctonia solani*. J. Agric. Chem. Environ..

[30] Nagle P., Pawar Y., Sonawane A., Bhosale S., More D. (2012). Synthesis and evaluation of antioxidant and antimicrobial properties of thymol containing pyridone moieties. Med. Chem. Res..

[31] Epps S.V., Harvey R.B., Byrd J.A., Petrujkić B.T., Sedej I., Beier R.C., Phillips T.D., Hume M.E., Anderson R.C., Nisbet D.J. (2015). Comparative effect of thymol or its glucose conjugate, thymol-β-D-glucopyranoside, on Campylobacter in avian gut contents. J. Environ. Sci. HealthPart. B.

[32] Dong L.M., Zhang M., Xu Q.L., Zhang Q., Luo B., Luo Q.W., Liu W.B., Tan J.W. (2017). Two new thymol derivatives from the roots of *Ageratina adenophora*. Molecules.

[33] Yang J., Li Y.C., Zhou X.R., Xu X.J., Fu Q.Y., Liu C.Z. (2018). Two thymol derivatives from the flower buds of *Lonicera japonica* and their antibacterial activity. Nat. Prod. Res..

[34] Bkhaitan M.M., Alarjah M., Mirza A.Z., Abdalla A.N., El-Said H.M., Hani S., Faidah H.S. (2018). Preparation and biological evaluation of metronidazole derivatives with monoterpenes and eugenol. Chem. Biol. Drug Des..

[35] El-Miligy M.M.M., Hazzaa A.A., El-Zemity S.R., Al-Kubeisi A.K. (2019). Synthesis of thymol derivatives as potential non-irritant antimicrobial and insecticidal agents. Curr. Bioact. Compd..

[36] Swain S.S., Paidesetty S.K., Padhy R.N. (2019). Synthesis of novel thymol derivatives against MRSA and ESBL producing pathogenic bacteria. Nat. Prod. Res..

[37] Kaur H., Lim S.M., Ramasamy K., Vasudevan M., Shah S.A.A., Narasimhan B. (2020). Diazenyl schiff bases: Synthesis, spectral analysis, antimicrobial studies and cytotoxic activity on human colorectal carcinoma cell line (HCT-116). Arab. J. Chem..

[38] Cui Z., Li X., Nishida Y. (2014). Synthesis and bioactivity of novel carvacrol and thymol derivatives containing 5-phenyl-2-furan. Lett. Drug Des. Discov..

[39] Wang K., Jiang S., Yang Y., Fan L., Su F., Ye M. (2019). Synthesis and antifungal activity of carvacrol and thymol esters with heteroaromatic carboxylic acids. Nat. Prod. Res..

[40] Javan A.J., Javan M.J. (2014). Electronic structure of some thymol derivatives correlated with the radical scavenging activity: Theoretical study. Food Chem..

[41] Ashraf Z., Rafiq M., Seo S.-Y., Kwon K.S., Babar M.M., Zaidi N.U. (2015). Kinetic and in silico studies of novel hydroxy-based thymol analogues as inhibitors of mushroom tyrosinase. Eur. J. Med. Chem..

[42] Sathe P.S., Rajput J.D., Gunaga S.S., Patel H.M., Bendre R.S. (2019). Synthesis, characterization, and antioxidant activity of thymol-based paracetamol analogues. Res. Chem. Intermed..

[43] Chen L.C., Lee T.H., Sung P.J., Shu C.W., Lim Y.P., Cheng M.J., Kuo W.L., Chen J.J. (2014). New thymol derivatives and cytotoxic constituents from the root of *Eupatorium cannabinum* ssp. *asiaticum*. Chem. Biodivers..

[44] Rajput J.D., Bagul S.D., Bendre R.S. (2017). Design, synthesis, biological screenings and docking simulations of novel carvacrol and thymol derivatives containing acetohydrazone linkage. Res. Chem. Intermed..

[45] Zhang Q.Q., Sun Z.Y., Feng X.Y., Chen R.J., Deng W., Tang Y.L., Guo Z.Y., Liu C.X., Chen J.F., Zou K. (2019). Thymol derivatives from the roots of *Eupatorium chinense* and their cytotoxic activities. Phytochem. Lett..

[46] Yu Y., Liu Y., Shi R., Zhang D., Li C., Shi J. (2020). New thymol and isothymol derivatives from *Eupatorium fortunei* and their cytotoxic effects. Bioorg. Chem..

[47] Chen J.J., Tsai Y.C., Hwang T.L., Wang T.C. (2011). Thymol, benzofuranoid, and phenylpropanoid derivatives: Anti-inflammatory constituents from *Eupatorium cannabinum*. J. Nat. Prod..

[48] Wang C., Zhang X., Wei P., Cheng X., Ren J., Yan S., Zhang W., Jin H. (2013). Chemical constituents from *Inula wissmanniana* and their anti-inflammatory activities. Arch. Pharm. Res..

[49] Nesterkina M., Kravchenko I. (2017). Synthesis and pharmacological properties of novel esters based on monoterpenoids and glycine. Pharmaceuticals (Basel).

[50] Mesquita B.M., do Nascimento P.G.G., Souza L.G.S., de Farias I.F., da Silva R.A.C., de Lemos T.L.G., Monte F.J.Q., Oliveira I.R., Trevisan M.T.S., da Silva H.C. (2018). Synthesis, larvicidal and acetylcholinesterase inhibitory activities of carvacrol/thymol and derivatives. Quim. Nova.

[51] Kurt B.Z., Gazioglu I., Dag A., Salmas R.E., Kayik G., Durdagi S., Sonmez F. (2017). Synthesis, anticholinesterase activity and molecular modeling study of novel carbamate-substituted thymol/carvacrol derivatives. Bioorg. Med. Chem..

[52] Zengin M., Genc H., Taslimi P., Kestane A., Guclu E., Ogutlu A., Karabay O., Gulçin I. (2018). Novel thymol bearing oxypropanolamine derivatives as potent some metabolic enzyme inhibitors—Their antidiabetic, anticholinergic and antibacterial potentials. Bioorg. Chem..

[53] Brotzman N., Xu Y., Graybill A., Cocolas A., Ressler A., Seeram N.P., Ma H., Henry G.E. (2019). Synthesis and tyrosinase inhibitory activities of 4-oxobutanoate derivatives of carvacrol and thymol. Bioorg. Med. Chem. Lett..

[54] Haddad J.G., Picard M., Bénard S., Desvignes C., Desprès P., Diotel N., El Kalamouni C. (2019). *Ayapana triplinervis* essential oil and its main component thymohydroquinone dimethyl ether inhibit Zika virus at doses devoid of toxicity in zebrafish. Molecules.

[55] Latacz G., Lubelska A., Jastrzębska-Więsek M., Partyka A., Marć M.A., Satała G., Wilczyńska D., Kotańska M., Więcek M., Kamińska K. (2019). The 1,3,5-triazine derivatives as innovative chemical family of 5-HT6 serotonin receptor agents with therapeutic perspectives for cognitive impairment. Int. J. Mol. Sci..

[56] Lindmark-Henriksson M., Isaksson D., Vaněk T., Valterová I., Högberg H.E., Sjödin K. (2004). Transformation of terpenes using a Picea abies suspension culture. J. Biotechnol..

[57] Babu L.H., Perumal S., Balasubramanian M.P. (2012). Myrtenal, a natural monoterpene, down-regulates TNF-a expression and suppresses carcinogeninduced hepatocellular carcinoma in rats. Mol. Cell. Biochem..

[58] Lingaiah H.B., Srinivasan P., Periyasamy B.M. (2012). Myrtenal attenuates diethylnitrosamine-induced hepatocellular carcinoma in rats by stabilizing intrinsic antioxidants and modulating apoptotic and anti-apoptotic cascades. Cell. Oncol..

[59] Rathinam A., Pari L., Chandramohan R., Sheikh B.A. (2014). Histopathological findings of the pancreas, liver, and carbohydrate metabolizing enzymes in STZ-induced diabetic rats improved by administration of myrtenal. J. Physiol. Biochem..

[60] Trytek M., Paduch R., Piet M., Koziel A., Kandefer-Szerszen M., Szajnecki Ł., Gromada A. (2018). Biological activity of oxygenated pinene derivatives on human colon normal and carcinoma cells. Flavour Frag. J..

[61] Barbuceanu S.F., Saramet G., Almajan G.L., Draghici C., Barbuceanu F., Bancescu G. (2012). New heterocyclic compounds from 1,2,4-triazole and 1,3,4-thiadiazole class bearing diphenylsulfone moieties. Synthesis, characterization and antimicrobial activity evaluation. Eur. J. Med. Chem..

[62] Uzgoren-Baran A., Tel B.C., Sarigol D., Ozturk E.I., Kazkayasi I., Okay G., Ertan M., Tozkoparan B. (2012). Thiazolo[3,2-b]-1,2,4-triazole-5(6H)-one substituted with ibuprofen: Novel non-steroidal anti-inflammatory agents with favorable gastrointestinal tolerance. Eur. J. Med. Chem..

[63] Liu X.H., Xu X.Y., Tan C.X., Weng J.Q., Xin J.H., Chen J. (2015). Synthesis, crystal structure, herbicidal activities and 3D-QSAR study of some novel 1,2,4-triazolo[4,3-a]pyridine derivatives. Pest. Manag. Sci..

[64] Lin G.S., Duan W.G., Yang L.X., Huang M., Lei F.H. (2017). Synthesis and antifungal activity of novel myrtenal-based 4-methyl-1,2,4-triazole-thioethers. Molecules.

[65] Lin G., Chen Z., Duan W., Wang X., Lei F. (2018). Synthesis and biological activity of novel myrtenal-derived 2-acyl-1,2,4-triazole-3-thione compounds. Chin. J. Org. Chem..

[66] Wanka L., Iqbal K., Schreiner P.R. (2013). The lipophilic bullet hits the targets: Medicinal chemistry of adamantane derivatives. Chem. Rev..

[67] Kapitsa I.G., Suslov E.V., Teplov G.V., Korchagina D.V., Komarova N.I., Volcho K.P., Voronina T.A., Shevela A.I., Salakhutdinov N.F. (2012). Search for new drugs. Synthesis and anxiolytic activity of 2-aminoadamantane derivatives containing monoterpene fragments. Pharm. Chem. J..

[68] Teplov G.V., Suslov E.V., Zarubaev V.V., Shtro A.A., Karpinskaya L.A., Rogachev A.D., Korchagina D.V., Volcho K.P., Salakhutdinov N.F., Kiselev O.I. (2013). Synthesis of new compounds combining adamantanamine and monoterpene fragments and their antiviral activity against influenza virus A(H1N1)pdm09. Lett. Drug Des. Discov..

[69] Suslov E.V., Ponomarev K.Y., Rogachev A.D., Pokrovsky M.A., Pokrovsky A.G., Pykhtina M.B., Beklemishev A.B., Korchagina D.V., Volcho K.P., Salakhutdinov N.F. (2015). Compounds combining aminoadamantane and monoterpene moieties: Cytotoxicity and mutagenic effects. Med. Chem..

[70] Ponomarev K.Y., Suslov E.V., Zakharenko A.L., Zakharova O.D., Rogachev A.D., Korchagina D.V., Zafar A., Reynisson J., Nefedov A.A., Volcho K.P. (2018). Aminoadamantanes containing monoterpene-derived fragments as potent tyrosyl-DNA phosphodiesterase 1 inhibitors. Bioorg. Chem..

[71] Ponomarev K.Y., Morozova E.A., Suslov E.V., Korchagina D.V., Tolstikova T.G., Volcho K.P., Salakhutdinov N.F. (2017). Synthesis and analgesic activity of 5,7- and 6-substituted diazaadamantanes containing monoterpene moieties. Chem. Nat. Compd..

[72] Gonda T., Bérdi P., Zupkó I., Fülöp F., Szakonyi Z. (2018). Stereoselective synthesis, synthetic and pharmacological application of monoterpene-based 1,2,4- and 1,3,4-oxadiazoles. Int. J. Mol. Sci..

[73] Nikitina L.E., Startseva V.A., Vakulenko I.A., Khismatulina I.M., Lisovskaya S.A., Glushko N.P., Fassakhov R.S. (2009). Synthesis and antifungal activity of compounds of the pinane series. Pharm. Chem. J..

[74] Silva A.C.R., Lopes P.M., Azevedo M.M.B., Costa D.C.M., Alviano C.S., Alviano D.S. (2012). Biological activities of *α*-pinene and *β*-pinene enantiomers. Molecules.

[75] Felipe C.F.B., Albuquerque A.M.S., de Pontes J.L.X., de Melo J.V., Rodrigues T.C.M.L., de Sousa A.M.P., Monteiro A.B., da Silva Ribeiro A.E., Lopes J.P., Menezes I.R.A. (2019). Comparative study of alpha- and beta-pinene effect on PTZ-induced convulsions in mice. Fund. Clin. Pharmacol..

[76] Salehi B., Upadhyay S., Orhan I.E., Jugran A.K., Jayaweera S.L.D., Dias D.A., Sharopov F., Taheri Y., Martins N., Baghalpour N. (2019). Therapeutic potential of *α*- and *β*-pinene: A miracle gift of nature. Biomolecules.

[77] Liao S., Shang S., Shen M., Xiaoping Rao X., Si H., Song J., Song Z. (2016). One-pot synthesis and antimicrobial evaluation of novel 3-cyanopyridine derivatives of (–)-*β*-pinene. Bioorg. Med. Chem. Lett..

[78] Liao S., Liu Y., Si H., Xiao Z., Fan G., Chen S., Wang P., Wang Z. (2017). Hydronopylformamides: Modification of the naturally occurring compound (–)-*β*-pinene to produce insect repellent candidates against *Blattella germanica*. Molecules.

[79] Nikitina L.E., Kiselev S.V., Startseva V.A., Lodochnikova O.A., Rakhmatullina A.A., Fedyunina I.V., Gilfanova I.R. (2019). New aspects of using biologically active thioterpenoids of pinane series. Russ. Chem. Bull. Int. Ed..

[80] Ye L., Zhang X., Xu Q., Cai Y., Gao W., Chen W. (2020). Anti-tumor activities and mechanism study of *α*-pinene derivative in vivo and in vitro. Cancer Chemoth. Pharm..

[81] Wang Y., Gu W., Shan Y., Liu F., Xu X., Yang Y., Zhang Q., Zhang Y., Kuang H., Wang Z. (2017). Design, synthesis and anticancer activity of novel nopinone-based thiosemicarbazone derivatives. Bioorg. Med. Chem. Lett..

[82] Wang Y., Wu C., Zhang Q., Shan Y., Gu W., Wang S. (2019). Design, synthesis and biological evaluation of novel *β*-pinene-based thiazole derivatives as potential anticancer agents via mitochondrial-mediated apoptosis pathway. Bioorg. Chem..

[83] Obieziurska M., Pacuła A.J., Długosz-Pokorska A., Krzemiński M., Janecka A., Ścianowski J. (2019). Bioselectivity induced by chirality of new terpenyl organoselenium compounds. Materials.

[84] Lin G.S., Ma C.H., Duan W.G., Cen B., Lei F.H., Yang Z.Q. (2014). Synthesis and biological activities of *α*-pinene-based dithiadiazoles. Holzforschung.

[85] Lee H.J., Hyun E.A., Yoon W.J., Kim B.H., Rhee M.H., Kang H.K., Cho J.Y., Yoo E.S. (2006). In vitro anti-inflammatory and anti-oxidative effects of *Cinnamomum camphora* extracts. J. Ethnopharmacol..

[86] Chen W., Vermaak I., Viljoen A. (2013). Camphor—A fumigant during the black death and a coveted fragrant wood in ancient Egypt and Babylon—A Review. Molecules.

[87] You C., Guo S., Zhang W., Yang K., Geng Z., Du S., Wang C., Deng Z. (2015). Identification of repellent and insecticidal constituents from *Artemisia mongolica* essential oil against *Lasioderma Serricorne*. J. Chem..

[88] Sokolova A.S., Yarovaya O.I., Shernyukov A.V., Gatilov Y.V., Razumova Y.V., Zarubaev V.V., Tretiak T.S., Pokrovsky A.G., Kiselev O.I., Salakhutdinov N.F. (2015). Discovery of a new class of antiviral compounds: Camphor imine derivatives. Eur. J. Med. Chem..

[89] Liu W., Ager D.J. (2005). Terpenes: The expansion of chiral pool. Handbook of Chiral Chemicals.

[90] Salakhutdinov N.F., Volcho K.P., Yarovaya O.I. (2017). Monoterpenes as a renewable source of biologically active compounds. Pure Appl. Chem..

[91] Yarovaya O.I., Sokolova A.S., Mainagashev I.Y., Volobueva A.S., Lantseva K., Borisevich S.S., Shtro A.A., Zarubaev V.V., Salakhutdinov N.F. (2019). Synthesis and structure-activity relationships of novel camphecene analogues as anti-influenza agents. Bioorg. Med. Chem. Lett..

[92] Zarubaev V.V., Pushkina E.A., Borisevich S.S., Galochkina A.V., Garshinina A.V., Shtro A.A., Egorova A.A., Sokolova A.S., Khursan S.L., Yarovaya O.I. (2018). Selection of influenza virus resistant to the novel camphor-based antiviral camphecene results in loss of pathogenicity. Virology.

[93] Sokolova A.S., Yarovaya O.I., Shernyukov A.V., Pokrovsky M.A., Pokrovsky A.G., Lavrinenko V.A., Zarubaev V.V., Tretiak T.S., Anfimov P.M., Kiselev O.I. (2013). New quaternary ammonium camphor derivatives and their antiviral activity, genotoxic effects and cytotoxicity. Bioorg. Med. Chem..

[94] Sokolova A.S., Yarovaya O.I., Baev D.S., Shernyukov A.V., Shtro A.A., Zarubaev V.V., Salakhutdinov N.F. (2017). Aliphatic and alicyclic camphor imines as effective inhibitors of influenza virus H1N1. Eur. J. Med. Chem..

[95] Silva E.T., Araújo A.S., Moraes A.M., Souza L.A., Lourenço M.C.S., Souza M.V.N., Wardell J.L., Wardell S.M.S.V. (2016). Synthesis and biological activities of camphor hydrazone and imine derivatives. Sci. Pharm..

[96] Kovaleva K.S., Zubkov F.I., Bormotov N.I., Novikov R.A., Dorovatovskii P.V., Khrustalev V.N., Gatilov Y.V., Zarubaev V.V., Yarovaya O.I., Shishkinad L.N. (2018). Synthesis of D-(+)-camphor-based *N*-acylhydrazones and their antiviral activity. Med. Chem. Commun..

[97] Sokolova A., Pavlova A., Komarova N., Ardashov O., Shernyukov A., Gatilov Y., Yarovaya O., Tolstikova T., Salakhutdinov N. (2016). Synthesis and analgesic activity of new *α*-truxillic acid derivatives with monoterpenoid fragments. Med. Chem. Res..

[98] Sokolova A.S., Yarovaya O.I., Bormotov N.I., Shishkina L.N., Salakhutdinov N.F. (2018). Discovery of a new class of inhibitors of Vaccinia Virus based on (–)-borneol from *Abies sibirica* and (+)-camphor. Chem. Biodivers..

[99] Artyushin O.I., Moiseeva A.A., Zarubaev V.V., Slita A.V., Galochkina A.V., Muryleva A.A., Borisevich S.S., Yarovaya O.I., Salakhutdinov N.F., Brel V.K. (2019). Synthesis of camphecene and cytisine conjugates using click chemistry methodology and study of their antiviral activity. Chem. Biodivers..

[100] Chernyshov V.V., Yarovaya O.I., Fadeev D.S., Gatilov Y.V., Esaulkova Y.L., Muryleva A.S., Sinegubova K.O., Zarubaev V.V., Salakhutdinov N.F. (2020). Single-stage synthesis of heterocyclic alkaloid-like compounds from (+)-camphoric acid and their antiviral activity. Mol. Divers..

[101] Mikláš R., Miklášová N., Bukovský M., Horváth B., Kubincová J., Devínsky F. (2014). Synthesis, surface and antimicrobial properties of some quaternary ammonium homochiral camphor sulphonamides. Eur. J. Pharm. Sci..

[102] Sokolova A.S., Baranova D.V., Yarovaya O.I., Baev D.S., Polezhaeva O.A., Zybkina A.V., Shcherbakov D.N., Tolstikova T.G., Salakhutdinov N.F. (2019). Synthesis of (1*S*)-(+)-camphor-10-sulfonic acid derivatives and investigations in vitro and in silico of their antiviral activity as the inhibitors of fi filovirus infections. Russ. Chem. Bull..

[103] Shi B., Luan D., Wang S., Zhao L., Tao L., Yuan Q., Wang X. (2015). Borneol-grafted cellulose for antifungal adhesion and fungal growth inhibition. RSC Adv..

[104] Vasconcelos R.M.C., Leite F.C., Leite J.A., Mascarenhas S.R., Rodrigues L.C., Piuvezam M.R. (2012). Synthesis, acute toxicity and anti-inflammatory effect of bornyl salicylate, a salicylic acid derivative. Immunopharm. Immunot..

[105] Sokolova A.S., Yarovaya O.I., Shtro A.A., Borisova M.S., Morozova E.A., Tolstikova T.G., Zarubaev V.V., Salakhutdinov N.F. (2017). Synthesis and biological activity of heterocyclic borneol derivatives. Chem. Heterocycl. Com..

[106] Xu X., Li J., Han S., Tao C., Fang L., Sun Y., Zhu J., Liang Z., Li F. (2016). A novel doxorubicin loaded folic acid conjugated PAMAM modified with borneol, a nature dual-functional product of reducing PAMAM toxicity and boosting BBB penetration. Eur. J. Pharm. Sci..

[107] Corrêa P.R.C., Miranda R.R.S., Duarte L.P., Silva G.D.F., Filho S.A., Okuma A.A., Carazza F., Morgado-Díaz J.A., Pinge-Filho P., Yamauchi L.M. (2012). Antimicrobial activity of synthetic bornyl benzoates against *Trypanosoma cruzi*. Pathog. Glob. Health..

[108] Silva A.T.M., Pereira V.V., de Almeida L.T.G., Ruiz A.L.T.G., de Carvalho J.E., Dias D.F., de Moreira M.E.C., Silva R.R., Duarte L.P. (2016). Synthesis and biological activity of borneol esters. Rev. Virtual Quim..

[109] Silva A.T.M., Pereira V.V., Takahashi J.A., Silva R.R., Duarte L.P. (2018). Microwave-assisted synthesis of borneol esters and their antimicrobial activity. Nat. Prod. Res..

[110] Setzer W.N., Setzer M.C., Bates R.B., Nakkiew P., Jackes B.R., Chen L., McFerrin M.B., Meehan E.J. (1999). Antibacterial hydroxycinnamic esters from *Piper caninum* from Paluma; North Queensland; Australia. The crystal and molecular structure of (+)-bornyl coumarate. Planta Med..

[111] Patil A., Ganguly S., Surana S. (2008). A systematic review of benzimidazole derivatives as an antiulcer agent. Rasayan J. Chem..

[112] Naga P.K., Kumar K.R. (2015). Green synthesis of benzimidazole derivatives: An overview of bulk drug synthesis. Int. J. Pharm. Tech. Res..

[113] Azam M.A., Suresh B. (2012). Biological activities of 2-mercaptobenzothiazole derivatives: A review. Sci. Pharm..

[114] Lokwani P., Nagori B.P., Batra N., Goyal A., Gupta S., Singh N. (2011). Benzoxazole: The molecule of diverse biological activities. J. Chem. Pharm. Res..

[115] Sokolova A.S., Yarovaya O.I., Semenova M.D., Shtro A.A., Orshanskaya I.R., Zarubaev V.V., Salakhutdinov N.F. (2017). Synthesis and in vitro study of novel borneol derivatives as potent inhibitors of the influenza A virus. Med. Chem. Commun..

[116] Borisova M.S., Yarovaya O.I., Semenova M.D., Tolstikova T.G., Salakhutdinov N.F. (2018). Antiulcerogenic activity of borneol derivatives. Russ. Chem. Bull..

[117] Kononova A.A., Sokolova A.S., Cheresiz S.V., Yarovaya O.I., Nikitina R.A., Chepurnov A.A., Pokrovskya A.G., Salakhutdinov N.F. (2017). *N*-Heterocyclic borneol derivatives as inhibitors of Marburg virus glycoprotein-mediated VSIV pseudotype entry. Med. Chem. Commun..

[118] Al-Farhan K.A., Warad I., Al-Resayes S.I., Fouda M.M., Ghazzali M. (2010). Synthesis, structural chemistry and antimicrobial activity of (–)-borneol derivative. Cent. Eur. J. Chem..

[119] Luo L., Li G., Luan D., Yuan Q., Wei Y., Wang X. (2014). Antibacterial adhesion of borneol-based polymer via surface chiral stereochemistry. ACS Appl. Mater. Interfaces.

[120] Sun X., Qian Z., Luo L., Yuan Q., Guo X., Tao L., Wei Y., Wang X. (2016). Antibacterial adhesion of poly(methyl methacrylate) modified by borneol acrylate. ACS Appl. Mater. Interfaces.

[121] Dorman H.J.D., Deans S.G. (2000). Antimicrobial agents from plants: Antibacterial activity of plant volatile oils. J. Appl. Microbiol..

[122] Mai L.M., Lin C.Y., Chen C.Y., Tsai Y.C. (2003). Synergistic effect of bismuth subgallate and borneol, the major components of Sulbogin^®^, on the healing of skin wound. Biomaterials.

[123] Meng L., Pan K., Zhu Y., Wei W., Li X., Liu X. (2018). Zwitterionic-based surface via the coelectrodeposition of colloid particles and tannic acid with bacterial resistance but cell adhesion properties. ACS Biomater. Sci. Eng..

[124] Xin Y., Zhao H., Xu J., Xie Z., Li G., Gan Z., Wang X. (2020). Borneol-modified chitosan: Antimicrobial adhesion properties and application in skin flora protection. Carbohydr. Polym..

[125] Li G., Zhao H., Hong J., Quan K., Yuan Q., Wang X. (2017). Antifungal graphene oxide-borneol composite. Colloids Surf. B Biointerfaces.

